# Building electrode/electrolyte interphases in aqueous zinc batteries via self-polymerization of electrolyte additives

**DOI:** 10.1093/nsr/nwae397

**Published:** 2024-11-11

**Authors:** Yaheng Geng, Wenli Xin, Lei Zhang, Yu Han, Huiling Peng, Min Yang, Hui Zhang, Xilin Xiao, Junwei Li, Zichao Yan, Zhiqiang Zhu, Fangyi Cheng

**Affiliations:** State Key Laboratory of Chemo/Biosensing and Chemometrics, College of Chemistry and Chemical Engineering, Hunan University, Changsha 410082, China; Greater Bay Area Institute for Innovation, Hunan University, Guangzhou 511300, China; State Key Laboratory of Chemo/Biosensing and Chemometrics, College of Chemistry and Chemical Engineering, Hunan University, Changsha 410082, China; Greater Bay Area Institute for Innovation, Hunan University, Guangzhou 511300, China; State Key Laboratory of Chemo/Biosensing and Chemometrics, College of Chemistry and Chemical Engineering, Hunan University, Changsha 410082, China; Greater Bay Area Institute for Innovation, Hunan University, Guangzhou 511300, China; State Key Laboratory of Chemo/Biosensing and Chemometrics, College of Chemistry and Chemical Engineering, Hunan University, Changsha 410082, China; Greater Bay Area Institute for Innovation, Hunan University, Guangzhou 511300, China; State Key Laboratory of Chemo/Biosensing and Chemometrics, College of Chemistry and Chemical Engineering, Hunan University, Changsha 410082, China; Greater Bay Area Institute for Innovation, Hunan University, Guangzhou 511300, China; State Key Laboratory of Chemo/Biosensing and Chemometrics, College of Chemistry and Chemical Engineering, Hunan University, Changsha 410082, China; Greater Bay Area Institute for Innovation, Hunan University, Guangzhou 511300, China; State Key Laboratory of Chemo/Biosensing and Chemometrics, College of Chemistry and Chemical Engineering, Hunan University, Changsha 410082, China; Greater Bay Area Institute for Innovation, Hunan University, Guangzhou 511300, China; State Key Laboratory of Chemo/Biosensing and Chemometrics, College of Chemistry and Chemical Engineering, Hunan University, Changsha 410082, China; Greater Bay Area Institute for Innovation, Hunan University, Guangzhou 511300, China; State Key Laboratory of Chemo/Biosensing and Chemometrics, College of Chemistry and Chemical Engineering, Hunan University, Changsha 410082, China; Greater Bay Area Institute for Innovation, Hunan University, Guangzhou 511300, China; State Key Laboratory of Chemo/Biosensing and Chemometrics, College of Chemistry and Chemical Engineering, Hunan University, Changsha 410082, China; Greater Bay Area Institute for Innovation, Hunan University, Guangzhou 511300, China; State Key Laboratory of Chemo/Biosensing and Chemometrics, College of Chemistry and Chemical Engineering, Hunan University, Changsha 410082, China; Greater Bay Area Institute for Innovation, Hunan University, Guangzhou 511300, China; State Key Laboratory of Advanced Chemical Power Sources, Engineering Research Center of High-efficiency Energy Storage (Ministry of Education), College of Chemistry, Nankai University, Tianjin 300071, China; Haihe Laboratory of Sustainable Chemical Transformations, Tianjin 300192, China

**Keywords:** aqueous zinc batteries, electrode/electrolyte interphases, interphase-forming additives, self-polymerization

## Abstract

Aqueous zinc batteries offer promising prospects for large-scale energy storage, yet their application is limited by undesired side reactions at the electrode/electrolyte interface. Here, we report a universal approach for the *in situ* building of an electrode/electrolyte interphase (EEI) layer on both the cathode and the anode through the self-polymerization of electrolyte additives. In an exemplified Zn||V_2_O_5_·nH_2_O cell, we reveal that the glutamate additive undergoes radical-initiated electro-polymerization on the cathode and polycondensation on the anode, yielding polyglutamic acid-dominated EEI layers on both electrodes. These EEI layers effectively mitigate undesired interfacial side reactions while enhancing reaction kinetics, enabling Zn||V_2_O_5_·nH_2_O cells to achieve a high capacity of 387 mAh g^−1^ at 0.2 A g^−1^ and maintain >96.3% capacity retention after 1500 cycles at 1 A g^−1^. Moreover, this interphase-forming additive exhibits broad applicability to varied cathode materials, encompassing VS_2_, VS_4_, VO_2_, α-MnO_2_, β-MnO_2_ and δ-MnO_2_. The methodology of utilizing self-polymerizable electrolyte additives to construct robust EEI layers opens a novel pathway in interphase engineering for electrode stabilization in aqueous batteries.

## Teaser

Glutamate additives, through their self-polymerization, construct a robust electrode/electrolyte interphase layer on both cathode and anode surfaces of aqueous zinc batteries, thereby significantly enhancing their overall performance.

## INTRODUCTION

Aqueous zinc batteries (AZBs) are highly intriguing candidates for large-scale energy storage on account of their intrinsic safety, environmental benignity, low cost and the high theoretical capacity (820 mAh g^−1^/5855 mAh cm^−3^) of the Zn anode [1,2]. Unfortunately, it is challenging to develop durable AZBs due to the undesirable side reactions that are initiated at the electrode/electrolyte interface [3,4]. Generally, the cathode materials suffer from complex parasitic reactions with water molecules, resulting in irreversible structural evolution and dissolution, and ultimately an unsatisfactory cycle life [5–9]. The Zn anode faces the issues of dendritic formation and side reactions (hydrogen evolution reaction (HER) and corrosion) at the anode/electrolyte interface, imposing the risk of short circuit and internal pressure increment in the battery [10–12]. Simultaneous alleviation of the above issues is a formidable challenge that must be addressed to realize the practical application of AZBs.

An effective and direct method to tackle the issues of interfacial side reactions in AZBs is to build a protective electrode/electrolyte interphase (EEI) on the surface of the electrode [13,14]. This approach has been well established in non-aqueous batteries, as the EEI layer can be easily derived from the decomposition of organic solvents or salts [15]. Nevertheless, it remains challenging to apply interphase engineering in aqueous electrolytes, particularly in the widely available, inexpensive ZnSO_4_ (ZSO) electrolyte, because water decomposition usually generates detrimental gases (H_2_, O_2_) instead of an effective protective layer, while SO_4_^2−^ is difficult to decompose at the restricted voltage windows of an aqueous electrolyte [15–18]. To attain the *in situ* EEI layer in aqueous electrolytes, various functional electrolytes have been rationally designed by the introduction of organic solvents and/or salts, such as highly concentrated electrolytes [9], deep eutectic electrolytes [17] and organic/aqueous electrolytes [19], which inevitably increase the cost (see the prices of common salts and solvents in Fig. S1 and Table S1) and/or decrease the safety and kinetics of aqueous batteries [15].

Alternatively, extensive efforts have been devoted to the construction of an EEI layer in the cost-effective ZSO electrolyte by the introduction of additives, which have impressive advantages in terms of cost, safety and kinetics [12,16]. The EEI layers primarily originate from the decomposition of additives whose compositions are usually uncontrollable [1,20,21]. Recently, organic monomer molecules have been developed as additives to construct polymeric EEI layers on Zn anodes through a polymerization reaction. These polymeric EEI layers usually feature abundant polar functional groups that could restrict the lateral movement of Zn^2+^, leading to uniform zinc deposition [16,20]. However, the research on organic monomer additives has primarily focused on the induction of the anode EEI layer, with scant attention directed towards the cathode side. This has consequently resulted in undesirable cycling performance of full cells. Therefore, the development of a universal interphase-forming additive that is capable of the *in situ* construction of well-defined and robust EEI layers on both the cathode and the anode of AZBs in a cost-effective ZSO electrolyte represents a highly desirable yet challenging objective.

**Figure 1.**
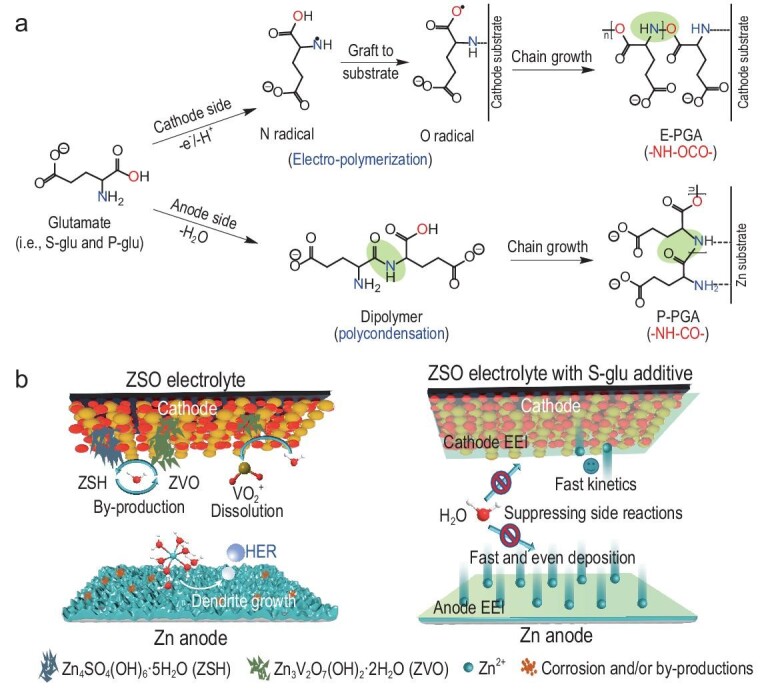
The design concept. (a) Proposed self-polymerization process of glutamate on the surface of cathode and Zn anode. (b) Schematic illustration of Zn||VOH cells when using ZSO electrolyte with/without glutamate additive.

Here, we introduce glutamate—a self-polymerizable molecule—as a universal interphase-forming additive to enable *in situ* formation of an EEI layer on both the cathode and the Zn anode of AZBs. We reveal that the glutamate additive undergoes a radical-initiated electrooxidation polymerization process on the cathode side, yielding an EEI layer that is dominated by electropolymerizing polyglutamic acid (denoted as E-PGA). On the anode side, the additive tends to polymerize via a polycondensation reaction, resulting in the formation of a robust EEI layer that is dominated by polycondensation-induced PGA (denoted as P-PGA). The *in situ*-formed EEI layers efficiently suppress the loss of active materials, accumulation of by-products and growth of Zn dendrite, while facilitating ionic diffusion and desolvation processes (Fig. 1b). Consequently, the Zn||V_2_O_5_·nH_2_O (VOH) cells with sodium glutamate (S-glu) demonstrated a high reversible capacity of 387 mA h g^−1^ at 0.2 A g^−1^, a superior rate performance of 171 mAh g^−1^ at 5 A g^−1^ and excellent cycling stability of 96.3% capacity retention after 1500 cycles at 1 A g^−1^. Moreover, this unique interphase-forming additive is applicable to various cathodes, including VO_2_, VS_2_, VS_4_, α-MnO_2_, β-MnO_2_ and δ-MnO_2_, highlighting its versatility in the interfacial engineering of electrode materials in cost-effective ZSO electrolytes.

## RESULTS AND DISCUSSION

The glutamate molecules could be polymerized via either a radical-initiated mechanism or a polycondensation reaction [22–25] to form PGA (Fig. 1a) on the surface of the electrode in aqueous solution, which makes it a promising interphase-forming additive to establish EEI layers in AZBs. Among glutamates, S-glu has recently been reported as an electrolyte additive to stabilize the Zn anode but its functional mechanism remains controversial. One view is that the S-glu additives could be adsorbed onto the Zn metal surface to redistribute Zn^2+^ flux and preclude water molecules [26], while another suggests that a protective EEI layer was formed on the Zn anode via the decomposition of S-glu [21]. Obviously, the self-polymerization ability of S-glu and its effects on the cathode have been completely overlooked in previous work.

**Figure 2.**
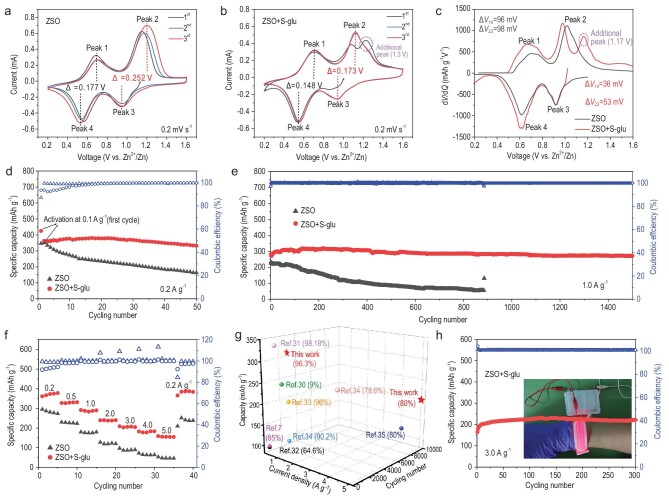
Electrochemical performance of the Zn||VOH cells. *CV* curves at 0.2 mV s^−1^ in the ZSO electrolyte (a) without and (b) with S-glu. (c) Initial d*Q*/d*V* curves in different electrolytes at 0.2 A g^−1^. Cycling performance at (d) 0.2 A g^−1^ and (e) 1.0 A g^−1^ and (f) rate performance in the ZSO electrolyte without/with S-glu. (g) Comparison of the cycling performance achieved in this work with reported VOH cathodes. The numbers in parentheses denote capacity retention rates. (h) Cycle stability of the pouch cell with the S-glu additives at 3 A g^−1^. Inset shows the optical photograph of the soft-package cell supporting the operation of a hand ring light.

To verify whether self-polymerizable S-glu could be used as a universal interphase-forming additive for aqueous batteries, we introduced a small amount of S-glu (∼0.3 M) into the cost-effective 2 M ZSO electrolyte and investigated its effectiveness in a typical aqueous Zn||VOH cell. The material characterization of VOH is shown in Fig. S2. The cyclic voltammetry (*CV*) curves (Fig. 2a) of the Zn||VOH cell in pure ZSO electrolyte exhibits two distinguishable redox peak pairs at ∼0.52/0.70 V (Peak 4/Peak 1) and 0.94/1.19 V (Peak 3/Peak 2), corresponding to the valence conversion of V to between +5/+4 and +4/+3, respectively [27]. After the introduction of S-glu additives, the *CV* curve showed an additional oxidation peak at 1.3 V in the initial cycle (Fig. 2b), which should be ascribed to the radical-initiated electro-polymerization of S-glu on the cathode surface [22,23]. Notably, this peak disappeared in the next two cycles, suggesting that the formed E-PGA film could passivate the electrode surface, thus preventing the continuous consumption of electrolyte. In addition, the *CV* curves in the second and third cycles showed two well-overlapped cathodic peaks at 0.95/1.12 V and two well-repeated anodic peaks at 0.55/0.69 V, indicating the excellent reversibility of the H^+^/Zn^2+^ (de)intercalation process [28]. Moreover, the *CV* curves of the S-glu-containing cell revealed narrower voltage gaps between the redox peaks compared with the S-glu-free one, demonstrating improved reaction kinetics [28]. Similar results can be found in the initial differential capacity (d*Q*/d*V*) plots (Fig. 2c), further suggesting the formation of the E-PGA film on the VOH cathode. Notably, although the electro-polymerization of S-glu would release protons, the pH value near the cathode part remained stable during cycling (Fig. S3). This is because this process only happens in the initial cycle and the –COO^−^ groups within the E-PGA-dominated EEI layer could capture the generated protons [10].

The cycle performances of the Zn||VOH cells in the ZSO electrolyte without and with the S-glu additives were also evaluated. As illustrated in Fig. 2d, the S-glu-free cell exhibited an initial capacity of 347 mAh g^−1^ at 0.2 A g^−1^, but only 46.5% of the original capacity remained after 50 cycles, which was attributable to the loss of active materials [29]. In contrast, the S-glu-containing cell delivered a high capacity of 381 mAh g^−1^ and achieved a high capacity retention of 91.8% after 50 cycles. When the current density was increased to 1 A g^−1^, the cell with S-glu still showed a high capacity of 318 mA h g^−1^ with 96.3% capacity retention after 1500 cycles, substantially outstripping that without the S-glu additive (Fig. 2e).

The rate capability of the VOH cathode was also improved with the S-glu additive (Fig. 2f and Fig. S4), delivering capacities of 386, 345, 297, 245, 213, 187 and 162 mAh g^−1^ at 0.2, 0.5, 1.0, 2.0, 3.0, 4.0 and 5.0 A g^−1^, respectively—far higher than those obtained in the S-glu-free electrolyte (46 mAh g^−1^ at 5 A g^−1^). When the current density was reset to 0.2 A g^−1^, the capacity of the S-glu-containing cell was recovered (395 mAh g^−1^), showing its good high-current tolerance. Moreover, the S-glu additive also enabled the Zn||VOH cell to realize excellent cycle stability at 5.0 A g^−1^, providing an initial capacity of 171 mAh g^−1^ with 81.3% capacity retention after 10 000 cycles (Fig. S5). This result far outperforms the previously reported VOH with ZSO electrolyte (Fig. 2g) [7,30–35], manifesting the superiority of the S-glu additive. Moreover, the S-glu worked well in the Zn||VOH pouch cell (Fig. 2h and Fig. S6), achieving a high capacity of 232 mAh g^−1^ at 3 A g^−1^, without observable capacity decay after 300 cycles. The practical efficacy of the pouch cell was also verified by powering red hand ring lights.

**Figure 3.**
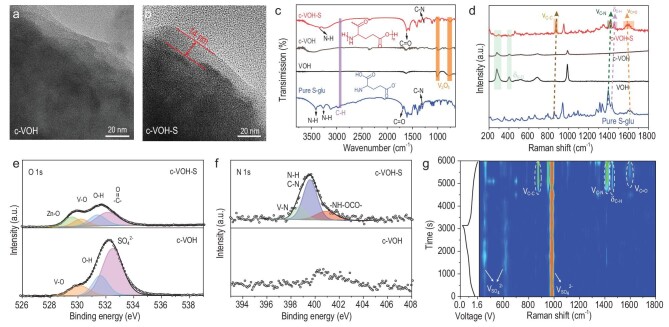
Formation of E-PGA-dominated cathode EEI. TEM images of the VOH cathode after two cycles in ZSO electrolyte (a) without and (b) with S-glu additive. (c) FTIR spectra and (d) Raman spectra of pristine and VOH cathodes after cycling in different electrolytes. (e) O 1s and (f) N 1s XPS spectra of VOH after cycling in different electrolytes for two cycles at 0.1 A g^−1^. All electrodes were collected at the fully charged state. (g) *In situ* Raman spectrum of the VOH cathode during the initial cycle.

To obtain a comprehensive understanding of the mechanisms underlying the enhanced performance, the morphological changes in the VOH cathode after cycling in different electrolytes was investigated via transmission electron microscopy (TEM). Similar to the pristine VOH cathode (Fig. S7), the cathode that was cycled in the ZSO electrolyte (denoted as c-VOH) maintained a smooth surface (Fig. 3a). In contrast, the cathode that was cycled in the S-glu-containing electrolyte (denoted as c-VOH-S) was covered by a uniform coating layer with a thickness of ∼14 nm, confirming the formation of a cathode EEI layer (Fig. 3b). This is also evidenced by the uniformly distributed N elements that were observed in the energy dispersive spectroscope (EDS) mapping images of the c-VOH-S (Fig. S8).

The chemical composition of the cathode EEI layer was then analysed by using Fourier transform infrared spectroscopy (FTIR) and Raman spectroscopy. As shown in Fig. 3c, the FTIR spectrum of c-VOH only exhibited the typical V–O stretching at 770 and 1005 cm^−1^ assigned to VOH [27], suggesting no formation of an EEI layer. In contrast, several new peaks appeared on the FTIR spectrum of the c-VOH-S, including the vibration stretching of the N–H of the –NH_2_ group (3330 cm^−1^), the typical peak of C=O stretching (1618 cm^−1^) and the signal of the vibration stretching of C–N (1275 cm^−1^) [16,36]. Notably, the peak positions of these functional groups are slightly different from those observed in the FTIR spectrum of pure S-glu, which could be ascribed to the formation of E-PGA, which is very consistent with the *CV* results. The Raman spectra were broadly in line with the FTIR observations. As shown in Fig. 3d, only the signals of V–O stretching (282 and 402 cm^−1^) were detected in c-VOH. By comparison, the characteristic peaks of C–C vibration (872 cm^−1^), C–N vibration (1421 cm^−1^), C–H stretching (1457 cm^−1^) and C=O vibration (1596 cm^−1^) [37,38] were observed in the Raman spectrum of c-VOH-S, which slightly differed from those of pure S-glu and further proved the formation of E-PGA.

The X-ray photoelectron spectroscopy (XPS) spectra of c-VOH and c-VOH-S were also collected. The V 2p (Fig. S9) and O 1s (Fig. 3e) XPS spectra of c-VOH only showed binding energies that corresponded to V–O, O–H and SO_4_^2−^ at 517.3/530.3, 531.6 and 532.7 eV, respectively, indicating no formation of the cathode EEI layer. In contrast, the C=O (532.1 eV) signal was detected in the O 1s spectrum of c-VOH-S (Fig. 3e) and the corresponding high-resolution N 1s spectrum showed strong peaks that related to the N–H/C–N (∼399.4 eV) and –NH–OCO– (∼400.9 eV) bonds (Fig. 3f), providing additional evidence of the formation of E-PGA [39,40]. Besides, the O 1s spectrum of c-VOH-S exhibited a signal at 529.78 eV that was ascribed to the Zn–O bond [35], suggesting an interaction between the E-PGA chain and the Zn^2+^. In the N 1s (Fig. 3f) and V 2p spectra (Fig. S9) of c-VOH-S, the observed characteristic peaks of the N–V bond (398.7 and 515.4 eV) suggested chemical adsorption of the formed EEI layer on the cathode surface [41]. This means that the EEI layer could adhere firmly to the cathode surface, which can accommodate the volume variations of the VOH electrode (Fig. S10), thereby ensuring its stability during long-term cycling (Figs S11 and S12).

To reveal the EEI layer initiation and evolution on the cathode side, the operando Raman measurement was employed to monitor the interfacial chemistry evolution of the VOH cathode during the initial cycle. As shown in Fig. 3g, only signals assigned to SO_4_^2−^ (458, 617 and 981 cm^−1^) were found during the initial discharging process and the appearance continued during the early charging process, suggesting no formation of the EEI layer. Notably, when the charge was 1.13 V, three obvious Raman bands at ∼873 (C–C vibration), ∼1419 (C–N vibration), ∼1459 (C–H stretching) and ∼1593 (C=O vibration) cm^−1^ emerged and remained prominent during the ensuing charging process, which could be assigned to the E-PGA that was derived from the electro-polymerization of S-glu [37,38]. This agrees well with the *CV* and d*Q*/d*V* curves of the S-glu-containing cell, which exhibited an additional oxidation peak that corresponded to the electro-polymerization of S-glu during the initial charging process. We also performed Raman (Fig. S13) and XPS (Fig. S14) measurements to detect the interfacial chemistry of the VOH after immersion in the S-glu-containing electrolyte for 24 h, which demonstrated that the EEI layer was not formed via the contact between the cathode and the electrolyte. These results revealed that a uniform and thin EEI layer dominated by E-PGA was formed on the surface of the c-VOH-S, which is expected to stabilize the VOH cathode.

**Figure 4.**
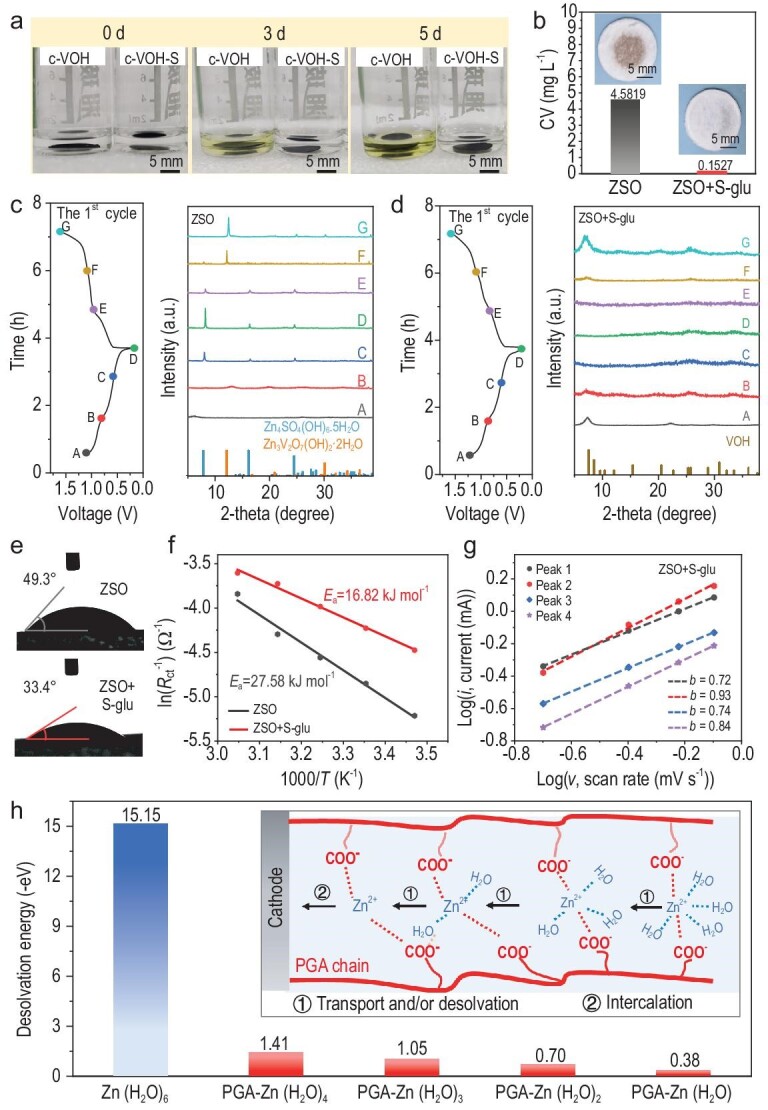
Effectiveness of EEI layer in inhibiting side reactions and improving the reaction kinetics. (a) Digital images of c-VOH and c-VOH-S soaked in ZSO solution. (b) Vanadium concentration in the electrolytes derived from the cycled cells. Insets show the digital images of the separator after cycling. *Ex situ* XRD patterns of the VOH cathode during the first cycle and the corresponding voltage profile at 0.1 A g^−1^ in (c) ZSO electrolyte and (d) ZSO + S-glu electrolyte. (e) Contact angle of the electrolytes on the cycled VOH cathodes. (f) Activation energies of the Zn||VOH cells after cycling in different electrolytes. (g) *CV* curves and (d) calculated *b*-values of the VOH cathode in the S-glu-containing electrolyte at different scan rates. (h) Contribution ratios of the capacitive capacities and diffusion-limited capacities of the VOH cathode. (i) Calculated desolvation energy barriers in different electrolytes. Inset is schematic illustration of the Zn^2+^ desolvation process in the PGA-dominated cathode EEI layer.

We then set out to verify the function of the EEI layer on inhibition of the dissolution of the VOH cathode. The formation of soluble yellow–brown VO_2_^+^ is known to be the main reason for the capacity fading of vanadium-based material in the ZSO electrolyte [42]. The c-VOH and c-VOH-S were soaked in the ZSO solution for 5 days and are visualized in Fig. 4a. After soaking, the ZSO solution with c-VOH changed from colorless to canary, while the solution with c-VO-S maintained colorless, suggesting that the formation of soluble VO_2_^+^ was effectively inhibited by the protection of the EEI. This was supported by the inductively coupled plasma–mass spectroscopy (ICP–MS) analysis (Fig. 4b), which indicated that the V concentration in the cycled S-glu-free electrolyte was 4.5819 mg L^−1^, but only 0.1527 mg L^−1^ of V was detected in the cycled S-glu-containing electrolyte. The glass-fiber separator from the cell with S-glu showed an invisible color change after cycling, while a yellow–brown precipitate that was indicative of vanadium dissolution was observed on the S-glu-free counterpart.

In addition to VO_2_^+^, other by-products such as Zn_4_SO_4_(OH)_6_·5H_2_O (ZSH) and Zn_3_V_2_O_7_(OH)_2_·2H_2_O (ZVO) would result in ineffective active material utilization and low electrical conductivity, thus impairing the cyclability of the VOH cathode [43]. Therefore, *ex situ* X-ray powder diffraction (XRD), scanning electron microscopy (SEM) and XPS were carried out to track the morphological and structural evolution of the VOH during cycling. The marked dots on the galvanostatic charge/discharge (GCD) curves (Fig. 4c and d, and Fig. S15) correspond to the states that were selected for the *ex situ* tests. For the S-glu-free electrode (Fig. 4c and Fig. S15a), the characteristic diffraction peaks of ZSH (8.07°, 16.22°, 24.43°) appeared when the discharge reached 0.5 V (Point D) and their intensity was gradually enhanced upon subsequent discharging, suggesting the formation and accumulation of ZSH. Upon charging, the characteristic peaks of ZSH gradually reduced and finally vanished at the fully charged state (Point G). Meanwhile, new diffraction peaks at 12.29°, 24.72° and 30.10° emerged after charging to 1.2 V (Point F), which can be indexed to ZVO. Similar phenomena were observed during the second cycle, implying the reversible formation and decomposition of ZSH and ZVO. Additionally, the peak intensity of these inert by-products significantly intensified during the second cycle, indicating a more pronounced formation of ZSH/ZVO. This is supported by the electrochemical impedance spectroscopy (EIS) measurement, which revealed that the interface resistance of the S-glu-free cell increased with cycling (Fig. S16a). Notably, with the addition of S-glu, no signals that were assigned to ZSH and ZVO were found throughout the *ex situ* XRD test (Fig. 4d and Fig. S15b), reflecting the effectiveness of the cathode EEI layer in suppressing the parasitic reactions. Accordingly, the interface resistance of the cell with S-glu decreased slightly after 3 cycles and remained almost unchanged after 100 cycles (Fig. S16b).

The identity of these by-products was confirmed by using SEM (Fig. S17) and EDS mapping (Fig. S18) images. After cycling in the S-glu-free electrolyte, a large amount of sheet-like by-products was observed on the electrode surface, at both fully charged and discharged states, corresponding to the formation of ZSH during discharging and ZVO during charging [43]. In stark contrast, the electrodes that were cycled in the S-glu-containing electrolyte showed a flat surface (Fig. S17), like a pristine electrode (Fig. S19). In addition, the S 2p XPS spectrum of c-VOH showed an intense SO_4_^2−^ signal (169.33 eV) at the fully discharged state [39], which became very weak at the fully charged state (Fig. S20), indicating the formation and decomposition of ZSH, while no obvious SO_4_^2−^ signal was observed in the S 2p spectra of c-VO-S at either states, further evidencing that the cathode EEI could suppress parasitic reactions.

Next, we evaluated the effects of the cathode EEI layer on the electrochemical kinetics. As shown in Fig. 4e, the contact angle of the S-glu-containing electrolyte on c-VO-S (33.4°) was smaller than that of the S-glu-free electrolyte on c-VO (49.3°). This disparity can be attributed to the rich polar groups (e.g. –COO^−^) that were present within the cathode EEI layer, which conferred superior surface wettability [44]. This enhanced wettability could reduce the interfacial free energy between the cathode and the electrolyte, thus improving the reaction kinetics [9]. To corroborate this, we measured the activation energy (*E*_a_) of the Zn^2+^ diffusion of the Zn||VOH cells in different electrolytes through temperature-dependent EIS (Fig. 4f and Fig. S21) [9]. Compared with the S-glu-free system, the cell with S-glu exhibited not only lower impedance at identical temperatures, but also a much lower *E*_a_ (16.82 vs. 27.58 kJ mol^−1^). Furthermore, the *CV* curves of the VOH with/without S-glu additives were collected at various scan rates to quantify the *b*-value and pseudocapacitance capacitive contribution for the Zn^2+^ storage (Fig. S22). Notably, all the *b*-values (Fig. 4g and Fig. S23) and capacitive contribution (Fig. S24) that were calculated for the S-glu-containing cell surpassed those of their S-glu-free counterparts, further implying the efficacy of the S-glu additive to promote the reaction kinetics [27,29,45,46].

Besides experimental characterizations, density functional theory (DFT) calculations were conducted in order to understand the enhanced kinetics. Generally, each Zn^2+^ was coordinated with six H_2_O molecules in the ZSO electrolyte, which necessitated a desolvation process before intercalation into the VOH cathode [6,44]. The desolvation energy barrier of solvated Zn^2+^ is as high as 15.15 eV (Fig. 4h), which would impede the kinetics of Zn^2+^ intercalation [44]. Notably, when Zn^2+^(H_2_O)_6_ passed through the E-PGA-dominated EEI layer, the coordinated water could be replaced by the carboxyl groups of E-PGA chains, forming a series of hydrated Zn^2+^ (PGA–Zn^2+^(H_2_O)*_n_, n* < 6) [44]. Importantly, the calculated desolvation energies of these hydrated Zn^2+^ structures are much lower than that of Zn^2+^(H_2_O)_6_, suggesting that the presence of a PGA-dominated cathode EEI layer could improve the kinetics of Zn^2+^ storage.

**Figure 5.**
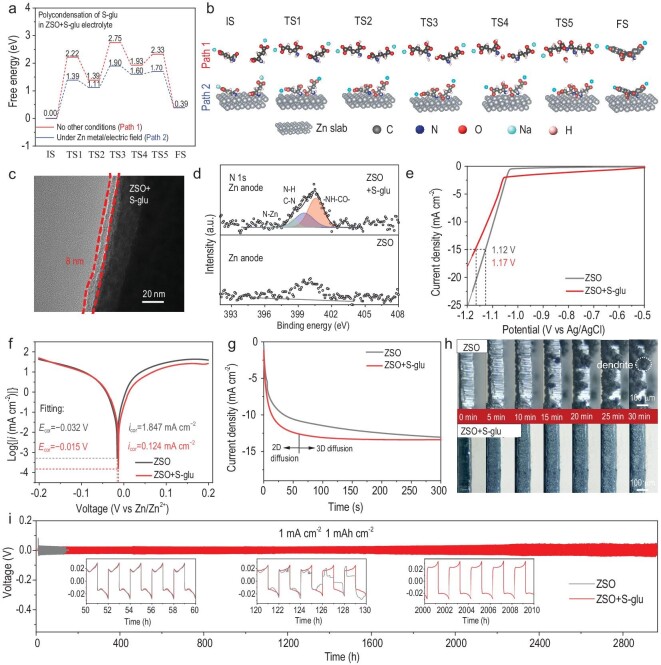
Effect of the S-glu additive on Zn anode. (a) DFT calculated free energy diagrams for the formation of P-PGA in ZSO + S-glu electrolytes under different conditions and (b) the corresponding structures of the initial state (IS), transition states (TS1–TS5) and final state (FS). (c) TEM image of the Zn anode after 10 cycles under 1 mA cm^−2^/1 mA h cm^−2^ in ZSO + S-glu electrolyte. (d) N 1s XPS spectra of Zn anodes after 10 cycles under 1 mA cm^−2^/1 mA h cm^−2^ in different electrolytes. (e) Linear sweep voltammetry curves of the Zn anode in ZSO with/without the S-glu additive using a three-electrode system at a scan rate of 10 mV s^−1^. (f) Linear polarization curves showing the corrosion on bare Zn anode in ZSO electrolytes with/without S-glu. (g) Chronoamperometric curves of the Zn anode for different electrolytes using symmetric Zn‖Zn cells at a negative overpotential of −150 mV. (h) *In situ* optical microscopy images of the Zn anodes during the Zn plating process in different electrolytes at 10 mA cm^−2^. (i) Cycling performance of symmetric Zn‖Zn cells at 1 mA cm^−2^/1 mA h cm^−2^ in different electrolytes.

In addition to stabilizing the VOH cathode, the S-glu additive could also induce an EEI layer on the surface of the Zn anode, which could improve its reversibility by suppressing dendrite growth and water-induced side reactions. In comparison with the cathode, the low overpotential of the Zn anode (∼50 mV) precludes the initiation of the radical-initiated electro-polymerization of S-glu [23]. Fortunately, S-glu molecules could undergo a polycondensation reaction (Fig. 1a) [24,25], offering an alternative pathway for yielding the anode EEI layer. To validate this inference, DFT-based transition state theory calculations were performed to investigate the polycondensation reaction of S-glu. As shown in Fig. 5a and b, the overall energy barrier of S-glu polycondensation in an aqueous electrolyte is as high as 2.75 eV, rendering it unlikely to occur spontaneously. However, this value is reduced to 1.90 eV in the presence of Zn and an electric field of ∼50 mV, suggesting a largely increased possibility of S-glu polycondensation on the Zn anode.

The formation of P-PGA on the Zn anode surface is supported by experimental characterizations. As shown by using TEM imaging, the Zn anode that was cycled in the ZSO electrolyte remained a bare surface, the same as the pristine one (Fig. S25), while the electrode that was cycled in the S-glu-containing electrolyte was covered by a uniform layer (Fig. 5c). The anode EEI was further investigated via XPS. In the high-resolution N 1s spectrum (Fig. 5d), the peak at 400.5 eV can be ascribed to the –NH–CO– group [31], suggesting the formation of a polycondensation-induced P-PGA. This signal differs from the electro-polymerization-induced E-PGA, which contains a –NH–OCO– group and shows a peak at 400.9 eV. The appearance of the N–Zn signal (398.6 eV) implies chemically adsorbed P-PGA (Fig. 5d), which enhances the adhesion on the Zn anode. Additionally, the N–H/C–N (399.5 eV) signals were observed in the N 1s spectrum (Fig. 5d) and the peaks of C–O (531.2 eV) and C=O (532.1 eV) appeared in the O 2s spectrum (Fig. S26a) [19,39], which could be assigned to P-PGA arising from S-glu polycondensation. The O 1s and S 2p spectra (Fig. S26) revealed two peaks at 529.8 and 168.9 eV, which could be assigned to Zn–O and SO_3_^2−^ [39], respectively, suggesting the presence of ZnSO_3_ in the anode EEI. On the contrary, no signal of organic species was detected on the Zn anode that was cycled in the electrolyte without S-glu. In the O 1s and S 2p spectra, the peaks at 531.7/532.5 and 169.8 eV are characteristic of O–H and SO_4_^2−^, which are identified as Zn_4_SO_4_(OH)_6_·*n*H_2_O, which is a typical by-product, on the anode side [39,47].

The formation of P-PGA-dominated EEI on the Zn anode is also revealed by using FTIR analysis. The FTIR spectrum of the Zn that was cycled in the S-glu-containing electrolyte exhibited three peaks at 3292, 2942 and 1602 cm^−1^ (Fig. S27), which correspond to the vibration stretching of N–H, C–H and C=O [19,39], respectively. Notably, the vibration band of C=O displayed a substantial red shift relative to that of E-PGA on the cathode side (1618 cm^−1^). We also studied the interfacial chemistry of the Zn anode after immersion in the S-glu-containing electrolyte (Figs S28 and S29), which suggests no EEI layer formation. These results reveal that the low overpotential on the Zn anode could initiate the polycondensation reaction of S-glu, thus yielding an organic–inorganic hybrid EEI layer that contained P-PGA and ZnSO_3_ on the anode surface.

To confirm the practical effect of the P-PGA/ZnSO_3_-containing EEI in stabilizing the Zn anode, the HER activity of different electrolytes was conducted by using a linear sweep voltammetry test. As indicated in Fig. 5e, the overpotential for the HER at 15 mA cm^−2^ was −1.17 V in the S-glu-containing electrolyte, which was lower than that in ZSO (−1.12 V), demonstrating the significant role of S-glu in inhibiting HER. Tafel plots reveal that the addition of S-glu significantly decreases the corrosion current (from 1.847 to 0.124 mA cm^−2^) while it increases the corrosion potential (from −0.032 to −0.015 V vs. Zn/Zn^2+^), reflecting the strong Zn corrosion resistance of the S-glu-induced anode EEI layer (Fig. 5f) [47]. This effect is also supported by the XRD (Fig. S30) and EDS mapping (Fig. S31) results, which indicated an invisible signal of by-product Zn_4_SO_4_(OH)_6_·0.5H_2_O with S-glu additive.

The Zn nucleation and growth behaviors were investigated by using chronoamperometry (CA) measurement under a constant overpotential of −150 mV for 300 s (Fig. 5g). The current densities in the ZSO electrolyte showed a continuous increase, representing the uncontrollable 2D diffusion of Zn^2+^ on the electrode surface [12]. When the S-glu was introduced into the ZSO electrolyte, a steady current density remained after a transiently increased current response within 58 s, suggesting a stable 3D diffusion process that is beneficial to the formation of dense and smooth Zn deposits [20]. Accordingly, the surface of the Zn anode that was cycled in the ZSO electrolyte became rough, while the one that was cycled in the S-glu electrolyte showed a relatively regular surface (Fig. S32). A similar phenomenon was also evidenced by the *in situ* optical microscopic observations (Fig. 5h), demonstrating the effectiveness of the S-glu additive on guiding the Zn^2+^ deposition.

The impact of the S-glu additive on the electrochemical performance of the Zn anode was further investigated in different cell configurations. As indicated in Fig. 5i and Fig. S33, the symmetric Zn||Zn cells that were operated in the ZSO electrolyte were short-circuited at 125 and 56 h under 1 mA cm^−2^/1 mAh cm^−2^ and 5 mA cm^−2^/5 mAh cm^−2^, respectively. After the addition of the S-glu additive, the lifespan of the symmetric cells was extended to 2950 h at 1 mA cm^−2^/1 mAh cm^−2^ and 1600 h at 5 mA cm^−2^/5 mAh cm^−2^. To evaluate the reversibility of the Zn plating/stripping, the coulombic efficiency (CE) of the asymmetric Zn||Ti cells was tested at a current density of 3 mA cm^−2^ with a capacity of 1.5 mAh cm^−2^. As exhibited in Figs S34 and S35, the Zn‖Ti cell without the S-glu displayed a CE of 98.17% and fluctuated to failure after only 58 cycles, which should have been caused by the dendrite growth and by-products accumulation. By comparison, the cell with S-glu delivered an average CE of 99.56% for 1400 cycles, clearly manifesting the effectiveness of the S-glu-induced EEI layer in stabilizing the Zn anode.

To further demonstrate the ability of the PGA-dominated EEI layer to boost the performance of both the cathode and the anode in AZBs, the cycling performance of the passivated-Zn||passivated-VOH cell and the symmetric passivated-Zn||passivated-Zn cell in pure ZSO electrolyte were supplemented. The passivated electrodes were collected from the cells after cycling in the ZSO + S-glu electrolyte and thus were covered by a PGA-dominated EEI layer already. Interestingly, the passivated-Zn||passivated-VOH cell with the ZSO electrolyte still displayed excellent cycle stability at 1.0 A g^−1^, providing a high capacity of 320 mAh g^−1^ with nearly 100% CE over 200 cycles (Fig. S36). Similarly, the symmetric passivated-Zn||passivated-Zn cells with the ZSO electrolyte stably operated over 130 h under 5 mA cm^−2^ and 5 mAh cm^−2^ (Fig. S37), which is a 2-fold improvement in cycle life compared with symmetric Zn|| Zn cells with the ZSO electrolyte (56 h). We also note that the performance of the cells that were based on the passivated electrodes that were cycled in the S-glu-free electrolyte was inferior to the performance of those that utilized pristine electrodes with S-glu present. This disparity can be ascribed to the influence of the S-glu additive on manipulating the electrolyte structure, thus improving battery performance (Fig. S38) [48]. Meanwhile, we utilized poly(glutamic acid) as an artificial coating layer on the Zn anode surface and then utilized this modified anode to assemble a symmetric cell that incorporated the ZSO electrolyte (Fig. S39). While this cell gained a modest improvement in cycle performance compared with the Zn||Zn cell with the same electrolyte, its performance remained inferior to that of the Zn||Zn cell that used the S-glu-containing electrolyte, further suggesting the advantages of the *in situ*-formed EEI layer.

**Figure 6.**
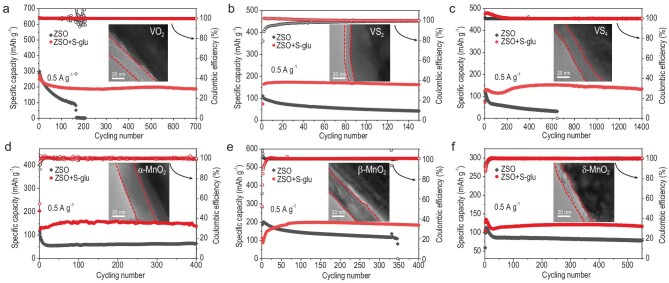
Universality of the S-glu additive for different cathode materials. Cycling performance of (a) Zn||VO_2_, (b) Zn||VS_2_, (c) Zn||VS_4_, (d) Zn||α-MnO_2_, (e) Zn||β-MnO_2_ and (f) Zn||δ-MnO_2_ in ZSO electrolytes without/with the S-glu additive at a current density of 0.5 A g^−1^. Insets show the TEM images of the cathodes after cycling in the ZSO + S-glu electrolyte.

To assess the universality of the S-glu additive, AZBs that were coupled with different cathodes, including VO_2_, VS_2_, VS_4_, α-MnO_2_, β-MnO_2_ and δ-MnO_2_, were investigated (see detailed material characterizations in Fig. S40). As shown in Fig. 6a, the Zn||VO_2_ cell with the S-glu additive retained a reversible capacity of ∼190 mAh g^−1^ with a high CE of 99.9% after 700 cycles at 0.5 A g^−1^, whereas the cell without S-glu rapidly failed after 128 cycles. The S-glu additive exerts an effect of stabilizing VS*_x_*, as revealed by the improved cycling performances of Zn||VS_2_ and Zn||VS_4_ cells with the S-glu-containing electrolyte (Fig. 6b and c). We also tested the effect of S-glu on addressing the dissolution issue of Mn-based cathode materials, which suffer from cycling instability due to Mn loss in aqueous electrolyte [3,4]. Although various strategies have been proposed to help to suppress the Mn^2+^ dissolution, the pre-addition of Mn^2+^ in electrolytes is still required for prolonged cycling. Nevertheless, the effect of pre-added Mn^2+^ would be reduced in practical batteries with lean electrolytes [49]. Interestingly, the presence of the S-glu additive can stabilize the MnO_2_ cathode even without the pre-addition of Mn^2+^. As shown in Fig. 6d–f, MnO_2_ cathodes with different phases operated stably for >400 cycles under 0.5 A g^−1^ in the ZSO electrolyte with the S-glu additive—far superior to those that were cycled in the S-glu-free electrolyte.

Similarly, the improved performance that these cathodes achieved in the S-glu-containing electrolyte can be attributed to the protection of the *in situ*-generated cathode EEI layer that was derived from the S-glu, as verified by using TEM (insets in Fig. 6), d*Q*/d*V* plots (Figs S41 and S42), FTIR (Fig. S43) and Raman spectroscopy (Fig. S44) measurements. It is noted that various cathodes undergo different activation processes during the initial cycling stages, which can be attributed to the unique chemical stability, reactivity, conductivity and microscopic characteristics of the materials [50]. In addition to S-glu, the incorporation of potassium glutamate (P-glu) as an electrolyte additive also leads to the formation of E-PGA on the VOH cathode and P-PGA on the Zn anode (Figs S45–S48), indicating that the PGA-dominated EEI formation is primarily attributed to the self-polymerization of glutamate. Consequently, the Zn||VOH full cell achieved a prolonged cycle life in the P-glu-containing electrolyte (Fig. S49). In addition, the beneficial effects of cations on the electrochemical properties are not significant, as evaluated by introducing 0.3 M K_2_SO_4_ or Na_2_SO_4_ into 2 M ZSO electrolyte (Fig. S50).

## CONCLUSION

We proposed a universal strategy for the *in situ* construction of EEI layers on both the cathode and the anode of AZBs through the self-polymerization of a glutamate additive during cycling. Using the Zn||VOH cell as a model system, we revealed that an E-PGA-dominated EEI layer that arose from the electro-polymerization of the glutamate additive could be firmly tied to the surface of the VOH cathode through the V–N bond, which not only suppresses the undesired cathode dissolution and side reactions, but also enhances the interfacial kinetics. Simultaneously, a robust EEI layer that comprised P-PGA/ZnSO_3_ was *in situ* generated on the anode surface via the polycondensation of glutamate, which inhibited the growth of Zn dendrites and the formation of by-products. As a result, the Zn||VOH cells with the S-glu additive delivered a high capacity of 386 mA h g^−1^ at 0.2 A g^−1^, a long lifespan of 1500 cycles with 96.3% capacity retention at 1 A g^−1^ and outstanding rate capability of 171 mA h g^−1^ at 5 A g^−1^—far surpassing the performance of reported VOH cathodes in the cost-effective ZSO electrolyte. Moreover, this interphase-forming additive was proven to be universally applicable to various cathode materials, including VO_2_, VS_2_, VS_4_, α-MnO_2_, β-MnO_2_ and δ-MnO_2_. This work opens up a new avenue for the *in situ* building of EEI layers in a cost-effective ZSO aqueous electrolyte via self-polymerizable additives, which will help to advance the development of high-performance aqueous rechargeable batteries.

## Supplementary Material

nwae397_Supplemental_File
