# Supplementary material for: Building electrode/electrolyte interphases in aqueous zinc batteries via self-polymerization of electrolyte additives
Source: Natl Sci Rev. 2024 Nov 11;12(1):nwae397. doi: 10.1093/nsr/nwae397 (PMC11740509; doi:10.1093/nsr/nwae397)
Supplement: nwae397_Supplemental_File [file nwae397_supplemental_file.pdf]

Supporting Information

Building Electrode/Electrolyte Interphases in Aqueous Zinc Batteries via Self-Polymerization of Electrolyte Additives

Yaheng Geng,[a] *Wenli Xin*[a] *Lei Zhang,*[a] *Yu Han,*[a] *Huiling Peng,*[a] *Min Yang,*[a] *Hui Zhang,*[a] *Xilin Xiao,*[a] *Junwei Li,*[a] *Zichao Yan,*[a] *Zhiqiang Zhu,**[*,a] *and Fangyi Cheng**[*,b]

a. State Key Laboratory of Chemo/Biosensing and Chemometrics, College of Chemistry and Chemical Engineering, Hunan University, Changsha, 410082, Hunan, China; Greater Bay Area Institute for Innovation, Hunan University, Guangzhou, 511300, Guangdong, China

b. State Key Laboratory of Advanced Chemical Power Sources, Engineering Research Center of High-efficiency Energy Storage (Ministry of Education), College of Chemistry, Nankai University, Tianjin 300071, China; Haihe Laboratory of Sustainable Chemical Transformations, Tianjin 300192, China

Email: zqzhu@hnu.edu.cn; fycheng@nankai.edu.cn

Materials and Methods

Preparation of electrolyte solutions

Zinc sulphate ($ZnSO_4$, 99%), sodium glutamate (S-glu, 98%), and potassium glutamate (P-glu, 99%) were purchased from Macklin, Bidepharm, and Aladdin respectively. The 2M $ZnSO_4$ aqueous solution with/without 50 g L^{-1} S-glu additive/55 g L^{-1} P-glu additive are empolyed as electrolytes. The water is purified by the Laboratory water purification system (Eco-S15Q, Hitech Instruments Co., Ltd).

Synthesis of $V_2O_5{\cdot}nH_2O$

The $V_2O_5{\cdot}nH_2O$ material was synthesized by the reaction of V_2O_5 (99.9.9%, Aladdin) powder with H_2O_2 at room temperature. Typically, 0.364 g of V_2O_5 powder and 1.6 mL of 30% H_2O_2 were added into 20 mL of deionized water to form a deep red solution. After resting for 10 h, the target $V_2O_5{\cdot}nH_2O$ was collected and washed thoroughly using water and then freeze-dried for 20 h.

Synthesis of VO_2

VO_2 nanorods were fabricated by a hydrothermal method. 1 g V_2O_5 powder was added into a 30 mL pure water and ethylene glycol (EG) mixture solution with a volum ratio of 3:2. After vigorously stirring for 2.5 hours, the suspension mixture was transfered into a 50 mL Teflon-lined autoclave and maintained at 180 ˚C for 5 hours. The obtained products was collected by centrifugation, and washed with pure water six times, then dried in a vacuum oven at 80 ˚C for 12 hours.

Synthesis of VS_2

VS_2 nanosheets were prepared via a simple hydrothermal method. Briefly, 2 mmol NH_4VO_3 was dissolved in 30 ml deionized water and 2 mL $NH_3.H_2O$ in a glass jar. Then, 15 mmol thioacetamide (TAA) was added in the homogeneous solution with continuous stirring at room temperature for 1 h. After that, the mixture was transferred to a 50 mL Teflon-lined autoclave at 180℃ for 20 h. Afterward, the

system was cooled down to room temperature naturally and the samples were washed with deionized water and ethanol thoroughly for five times, respectively. The final product was dried at 60°C for 8 h in vacuum.

Synthesis of VS_4

In a typical process, 0.35 g of ammonium metavanadate (NH_4VO_3) was added into 30 mL of deionized water. Then the suspension was heated to 60 °C, forming a clear pale-yellow solution. Then, 30 mL of 0.6 M thioacetamide (TAA) dissolved in ethylene glycol (EG) was added in the above solution. Finally, the homogeneous solution was transferred into a Teflon-lined autoclave with a capacity of 100 mL. The autoclave was heated at 160 °C for 16 h. The precipitate product was rinsed with a centrifuge by deionized water and ethanol for five times and dried in a vacuum oven at 60 °C for 12h.

Synthesis of α-MnO_2

The α-MnO_2 nanorods were synthesized using a hydrothermal method. 45 mL of $MnSO_4 \cdot H_2O$ (0.15M) solution and 45 mL $KMnO_4$ (0.15M) solution were mixed under stirring. The mixture was then heated 160 ℃ for 12 h in a 100 ml Teflon-lined autoclave. After the reaction, the precipitation was collected and washed with deionized water, and then vacuum dried at 80 ℃ for 12h.

Synthesis of β-MnO_2

β-MnO_2 nanorods were synthesized by a hydrothermal method following previously reported procedures. In a typical synthesis, 30 mL $KMnO_4$ (0.1 M) and 30 mL $MnSO_4 \cdot H_2O$ (0.6 M) were mixed under continuous stirring for 30 min at room temperature. The mixture was loaded into a 100 ml Teflon-lined autoclave and maintained at 140 °C for 12 h. The obtained product was centrifuged, washed thoroughly using water and absolute ethyl alcohol, and vacuum dried at 80 °C for 10 h.

Synthesis of δ-MnO_2

δ-MnO_2 was synthesized by hydrothermal method. 40 mL $KMnO_4$ (0.6 M) and 40 mL $MnSO_4 \cdot H_2O$ (0.1 M) were mixed under continuous stirring for 30 min at room temperature. The solution was put into a 100 ml Teflon-lined autoclave and heated at 160 °C for 12h. The solution was then cooled to room temperature. δ-MnO_2 powder was obtained through the centrifuge, washed with pure water three times, and dried at 60 °C overnight.

Materials characterizations

Fourier transform infrared spectroscopy (FTIR) was recorded using KBr pellets on a BRUKER TENSOR II (FTS6000) in unit of cm^{-1}. Raman spectra were recorded using a Raman microscope (Renishaw invia) with a 532 nm diode laser. Scanning electron microscopy (SEM) images were obtained using TESCAN MIRA3. Transmission electron microscopy (TEM) images were acquired on Talos f200i with an electron acceleration energy of 200 kV. X-ray diffraction (XRD) patterns were recorded by PANalytical Empyren X-ray diffractometer with Cu K_α radiation (45kV and 40mA). X-ray photoelectron spectroscopy (XPS) measurements were conducted on an X-ray photoelectron spectrometer (Thermo Fischer, ESCALAB Xi+) under a vacuum of 8×10^{-10} Pa (the C 1s peak at 284.8 eV as reference). Inductively coupled plasma emission spectrometry (ICP-OES) was characterized by the Agilent ICP-OES730. The V concentration in electrolyte after 2 cycles at 0.1 A g^{-1} were test by ICP-OES, where the electrolyte in membrane were extracted by 0.5 mL concentrated HNO_3 and diluted to 10 mL with H_2O. For characterizing of EEI layer, the various cathodes were activated two cycle at 0.1 A g^{-1}, and then the morphology and chemical composition of cathodes was investigated by XPS, TEM, FTIR, Raman spetrum. In order to avoid interference from the electrolyte, the surface of the activated cathodes were cleaned by water and ethanol before test.

Electrochemical measurements

Coin-type cells (CR2032) were assembled for symmetric Zn||Zn cells, Zn||Ti half cells with the glass fiber as the separator. To fabricate the cathodes, slurries were first prepared by mixing the active material, commercial conductive paste and poly(vinylidene fluoride) (PVDF) binder with a weight ratio of 7:2:1 in N-methyl-2-pyrrolidone (NMP, Macklin, 99.5%). These slurries were then coated onto Ti foil (for VOH, VO_2, VS_2, and VS_4) or stainless steel (for α-MnO_2, β-MnO_2, and δ-MnO_2), and then dried at 80 °C for 12 h. Full cells were assembled using Zn plate as anode, VOH as cathode, glass fiber as separator in coin-type cells (CR2032) and pouch cells (6 cm × 8 cm). In addition, some other cathode materials including VO_2, VS_2, VS_4, α-MnO_2, β-MnO_2, and δ-MnO_2 were also used to assembled coin-type full cells (CR2032). For the coin-type cells using Ti foil, the diameter of electrode sheet and separator is 10 mm and 19 mm, respectively. For the coin-type cells using stainless steel, the diameter of electrode sheet and separator is 15 mm and 19 mm, respectively. The electrochemical impedance spectroscopy (EIS) was measured with a frequency range from 100 kHz to 0.05 Hz and an AC amplitude of 10 mV using an Admiral instruments electrochemical workstation for symmetric Zn||Zn cells and full cells. Cyclic voltammetry (CV) tests were conducted on Admiral instruments electrochemical workstation. The chronoamperometry (CA) was performed using symmetric Zn||Zn cells on Admiral instruments electrochemical workstation. Linear polarization was measured by scanning between −0.2 and 0.2 V (vs. the open circuit potential) at 1 mV s^{-1} using symmetric Zn||Zn cells. The Coulombic efficiencies (CE) of the Zn||Ti cells were measured at 3 mA cm^{-2} with a fixed cycling capacity of 1.5 mA h cm^{-2}. Linear sweep voltammetry was conducted in a three-electrode configuration at 10 mV s^{-1} (Pt foil as the working electrode and counter electrode, Ag/AgCl as the reference electrode). The charge/discharge experiments were performed on a Land CT3001AU battery test system at room temperature.

Theoretical calculation

Quantum chemistry (QC) calculation was performed with a Gaussian16 software to investigate the interaction between ions or molecules. B3LYP functional was used as it is robust for both main group elements and transition elements. D3(BJ) dispersion correction was used to improve the precision of weak interactions. 6-311+G (d, p) basis set was used for C, H, O and N atoms while SDD basis set was used for Zn atoms. Molecules were optimized and had no imaginary frequency in vibration analysis. In the potential energy curve calculations, the two segments are optimized, respectively. The universal solvation model SMD was used to consider the aqueous environment. The desolvation energy value can be obtained by equation:

$$E = E(i\text{-}xH_2O) - E(i) - xE(H_2O)$$

where E is the desolvation energy of compound (Zn-6H_2O or Zn-PGA-xH_2O), E(i-xH_2O) is the energy of compound (Zn-6H_2O or Zn-PGA-xH_2O) aquo-complex, E(i) is the energy of compound (Zn^{2+} or Zn-PGA), E(H_2O) is the energy of H_2O.

The density functional theory (DFT) calculations were performed using a Dmol3 module of Material Studio 2020. The generalized gradient approximation (GGA) method with Perdew-Burke-Ernzerh of (PBE) function was employed to describe the interactions between core and electrons. The force and energy convergence criterion were set to 0.002 Ha $Å^{-1}$ and 10^{-5} Ha, respectively. Solvation energy corrections were calculated in water with the SMD continuum solvation model based on the gas-phase optimized geometries. The voltage of 50 mV was added to the Zn electrode in the all of reaction.

When the optimization was completed, the transition states were located utilizing the well-known linear synchronous transit (LST) and quadratic synchronous transit (QST) methods. After the LST/QST calculations, the frequency calculations were performed. A true transition state from LST/QST calculations was confirmed by a single negative frequency. The free energy corrections were accomplished with dmol3 at a temperature of 298.15K.

Results and Discussion

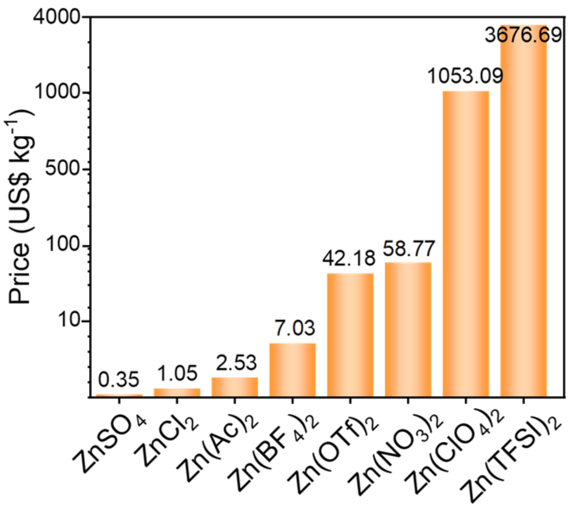

Figure S1. Price of various Zn salts.

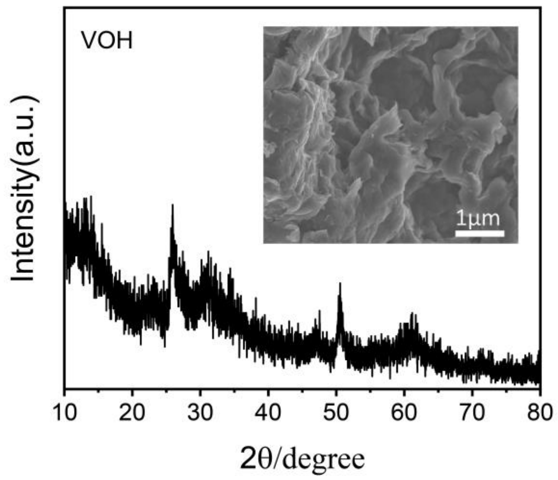

Figure S2. XRD pattern and SEM image (inset) of the as-prepared VOH cathode material.

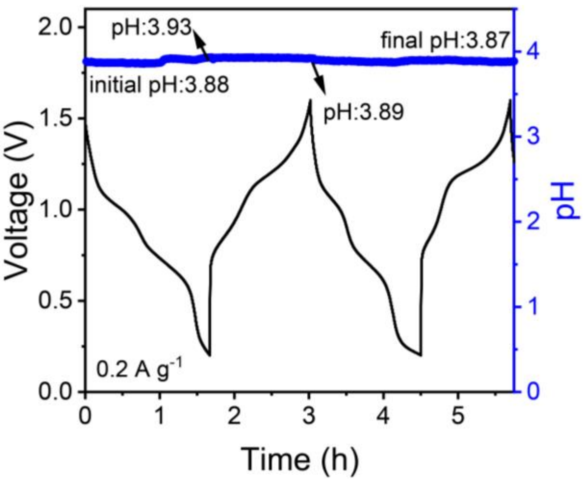

Figure S3. Variation of pH value of the Zn||VOH cell with the ZSO+S-glu electrolyte during the first two cycles.

According to the CV (Figure 2b) and dQ/dV (Figure 2c) curves of the Zn||VOH cells, the in-situ electro-polymerization of S-glu only occurs in the initial charging process, which could yield a E-PGA dominated EEI layer with a thickness of ~14 nm (Figure 3b). This EEI layer could passivate the electrode surface, which could in turn prevent the continuous electro-polymerization of S-glu. For this reason, only limited protons would be released during the in-situ electro-polymerization process of S-glu even though 0.3 mol L^{-1} of S-glu was added in the electrolyte. In addition, the E-PGA dominated EEI layer contains a large amount of $-COO^{-}$ groups, which could serve as a pH buffer zone by capturing the generated protons, thus mitigating the pH variation during the electro-polymerization of S-glu. To validate this inference, an in-situ pH detection configuration was introduced to monitor the pH variation near the cathode part. As shown in Figure S3, the pH value remains stable during the initial two cycles, which can be attributed to the evident buffer effect of $-COO^{-}$ in PGA structure.

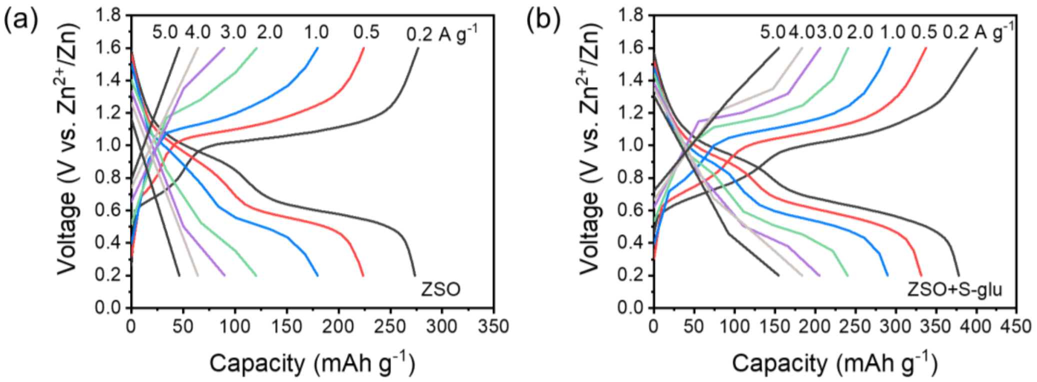

Figure S4. Typical discharge/charge profiles of the Zn||VOH cells in different electrolytes at various current densities.

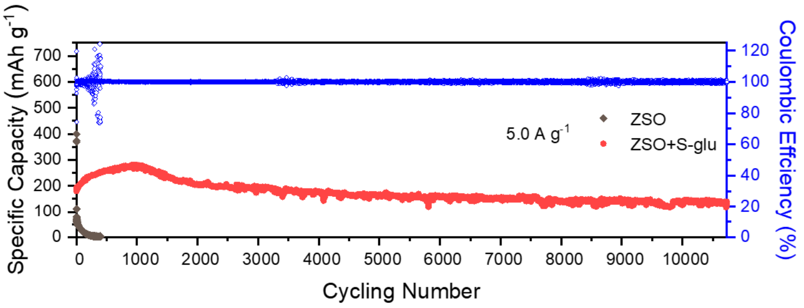

Figure S5. Cycling performance of the Zn||VOH cell in ZSO electrolyte with/without S-glu additive at 5 A g^{-1}.

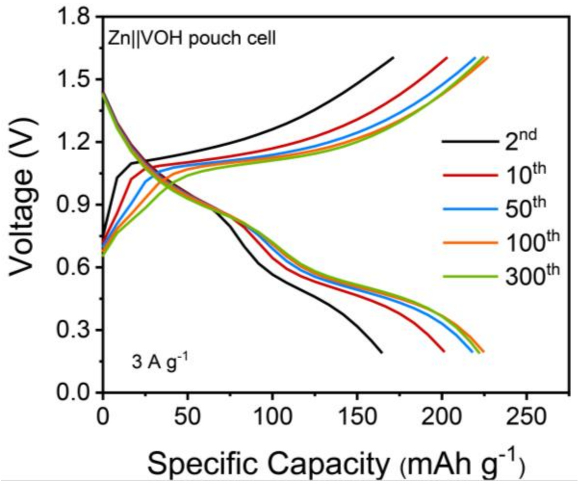

Figure S6. Typical discharge/charge profiles of the pouch cell in the S-glu containing electrolyte at 3 A g^{-1}.

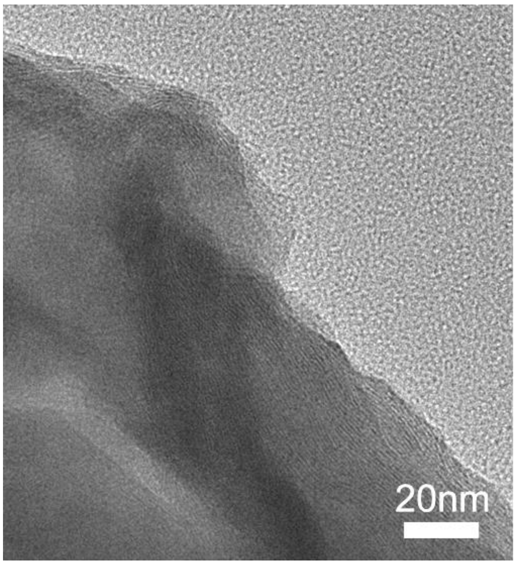

Figure S7. TEM image of the pristine VOH cathode.

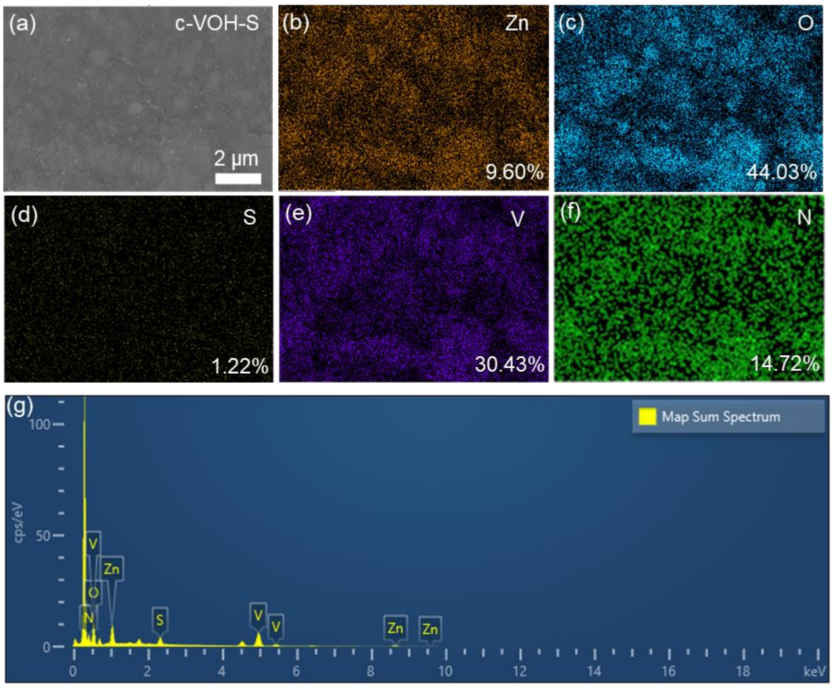

Figure S8. (a) SEM image and corresponding energy dispersive spectrum with the elemental maps of (b) Zn, (c) O, (d) S, (e) V, (f) N, and (g) map sum spectrum of the VOH electrodes after cycling in ZSO electrolyte with S-glu additive for two cycles.

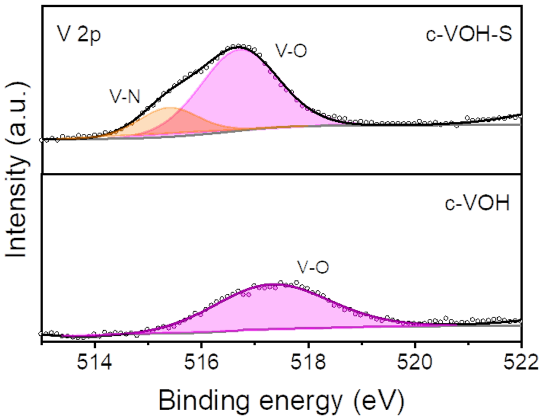

Figure S9. V 2p XPS spectra of the VOH cathode after cycling in different electrolytes for 2 cycles at 0.1 A g^{-1}.

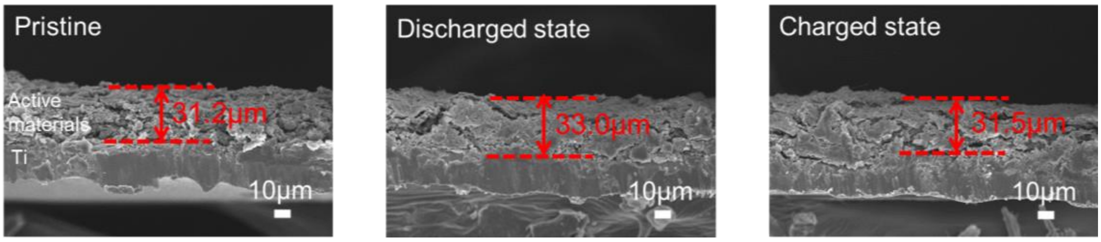

Figure S10. SEM images of the VOH electrodes at different states: a) pristine, b) fully discharged, c) fully charged. The fully charged and discharged electrodes were collected after cycling in the S-glu containing electrolyte for 25 cycles. All these electrodes were selected from the same batch to ensure close thickness of the pristine electrodes.

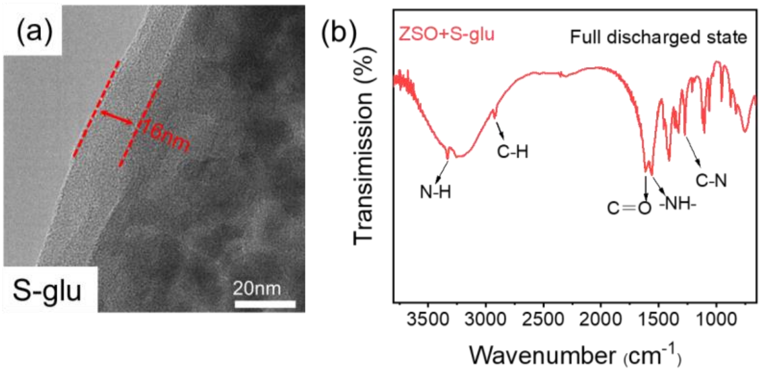

Figure S11. (a) TEM image of the VOH cathode under full discharged state in the second cycle (0.1 A g^{-1}) in ZSO+S-glu electrolyte. (b) FTIR spectra of VOH cathodes under full discharged state in the second cycle (0.1 A g^{-1}) in ZSO+S-glu electrolytes.

Under the fully discharged state, the c-VOH-S was covered by a uniform coating layer with a thickness of ~16 nm, mirroring its appearance under the fully charged state. This underscores the stability of the EEI layer. Furthermore, the FTIR spectrum of the c-VOH-S under the fully discharged states exhibited characteristic peaks of N-H stretching (~3331 cm^{-1}), C=O stretching (~1616 cm^{-1}), and of C-N stretching (~1273 cm^{-1}), further confirming the presence of the EEI layer. These results clearly demonstrate that the robust adherence of the EEI layer to the cathode surface, underscoring its reliability and stability during long-term cycling.

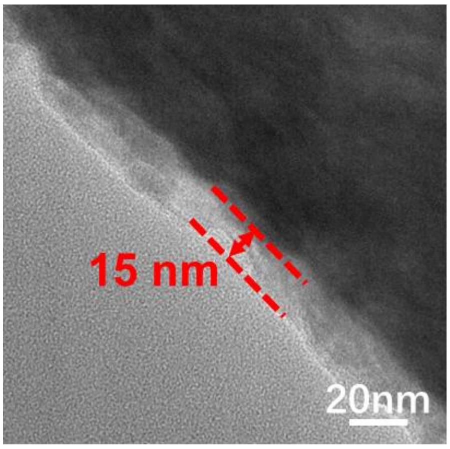

Figure S12. TEM image of the VOH cathode after cycling in the S-glu containing electrolytes (2 cycles at 0.1 A g^{-1} and then 25 cycles at 1 A g^{-1}).

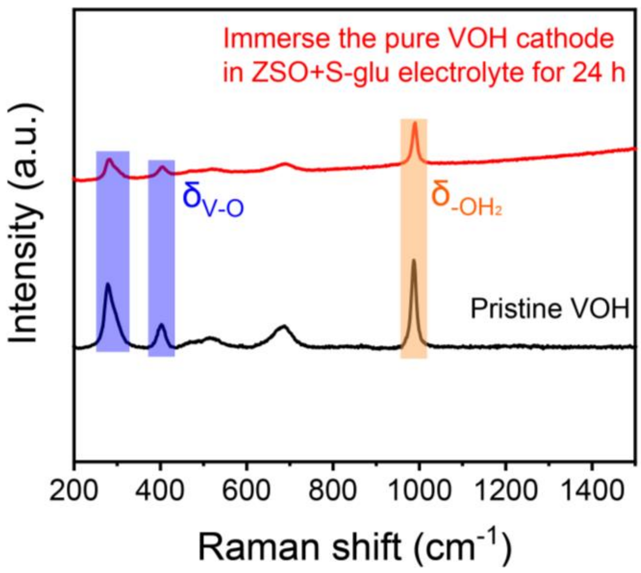

Figure S13. Raman spectra of the pristine VOH cathode and the VOH cathode after immersing in ZSO+S-glu electrolyte for 24 h.

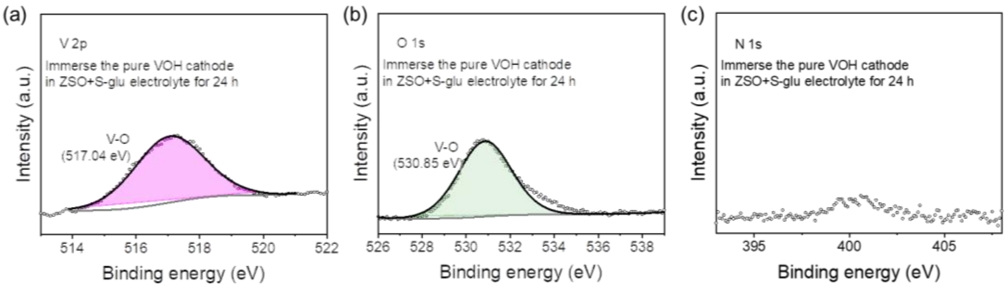

Figure S14. (a) V 2p (b) O 1s, and (c) N1s XPS spectra of the VOH cathode after immersing in ZSO+S-glu electrolyte for 24h.

The V 2p and O 1s XPS spectra of VOH after soaking only showed the binding energies of V-O at 517.04/530.85 eV, demonstrating that the cathode EEI layer cannot be formed by simply soaking.

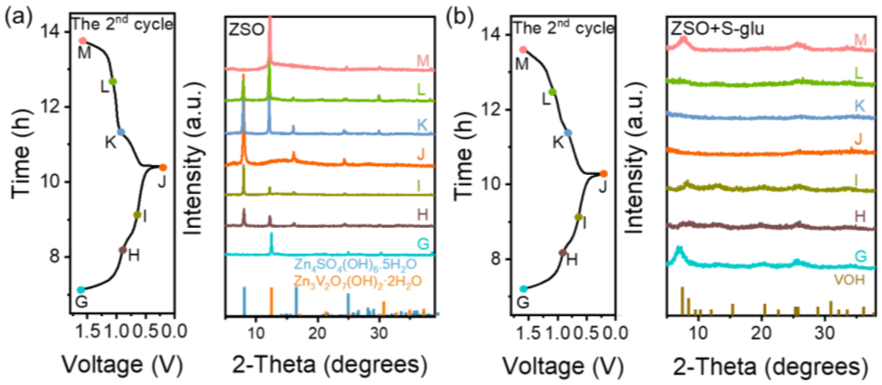

Figure S15. Ex-situ XRD patterns of the VOH cathode during the second cycle and the corresponding voltage profile at 0.1 A g^{-1} in (a) ZSO electrolyte and (b) ZSO+S-glu electrolyte.

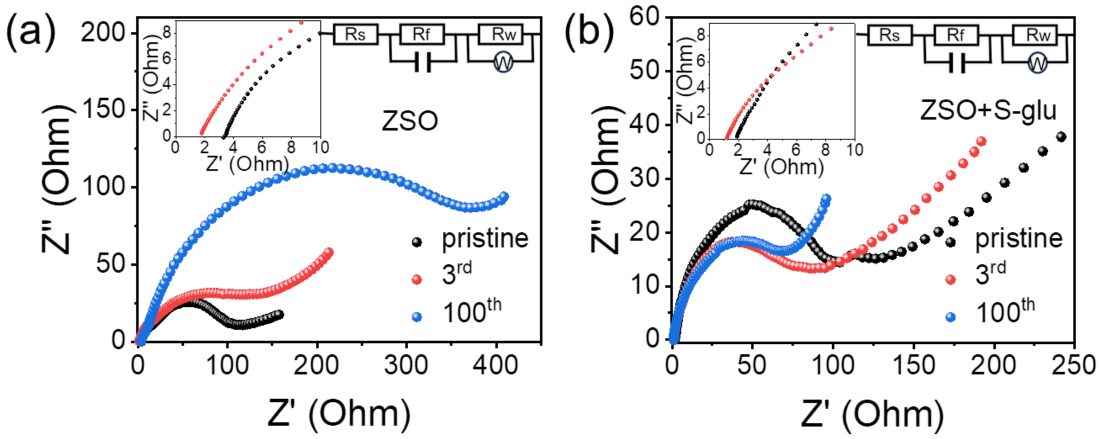

Figure S16. EIS spectra of the VOH cathode after cycling in the ZSO electrolytes (a) without and (b) with S-glu additive for different cycles. Insets are the partial enlargement (left part) and fitting circuit diagram (right part) of the EIS spectra.

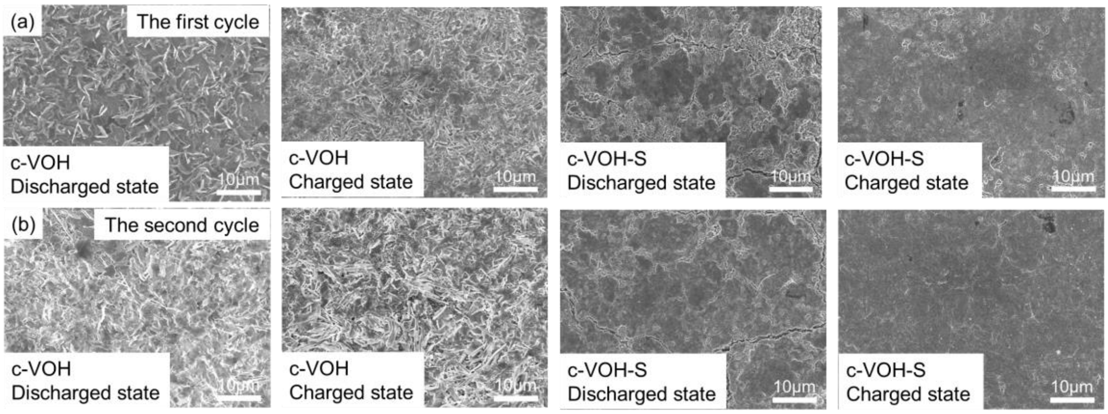

Figure S17. SEM images of the VOH cathodes after cycling in the ZSO electrolyte without/with S-glu additive under different charge/discharge states for (a) the first and (b) second cycle.

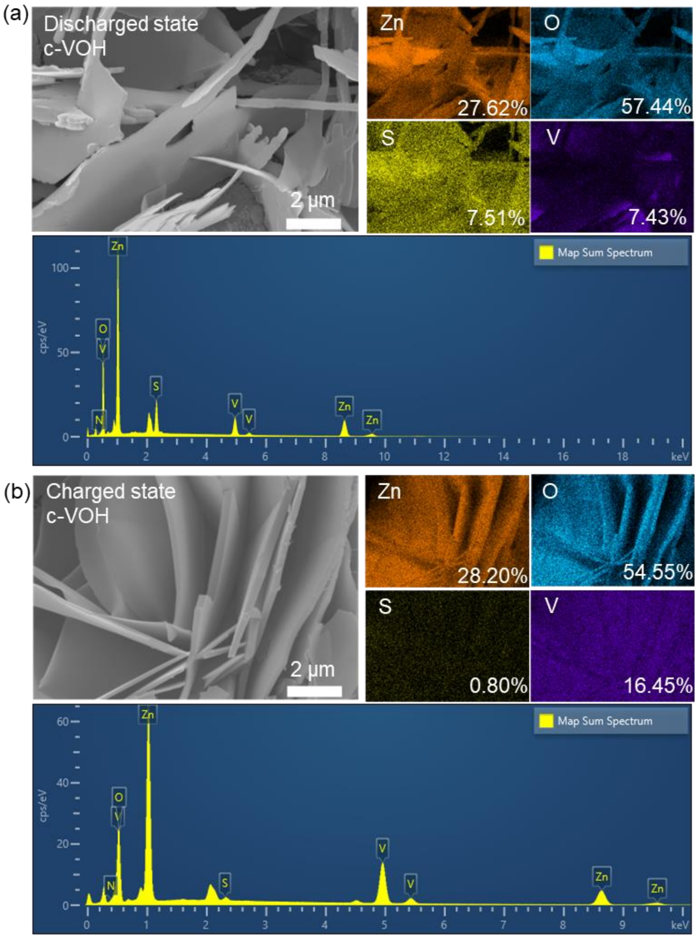

Figure S18. SEM image and corresponding energy dispersive spectrum with the elemental maps of Zn, O, S, and V of the VOH electrodes after cycling in ZSO electrolyte for two cycles at the (a) discharged and (b) charged state.

Here, it was found that the ratio of Zn and S is about 1/4 under discharged state and the ratio of Zn and V is about 3/2 under charged state, which should be assigned to ZSH and ZVO, respectively.

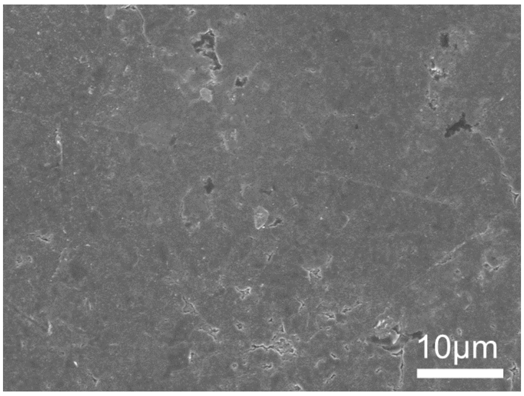

Figure S19. SEM image of the pristine VOH electrode.

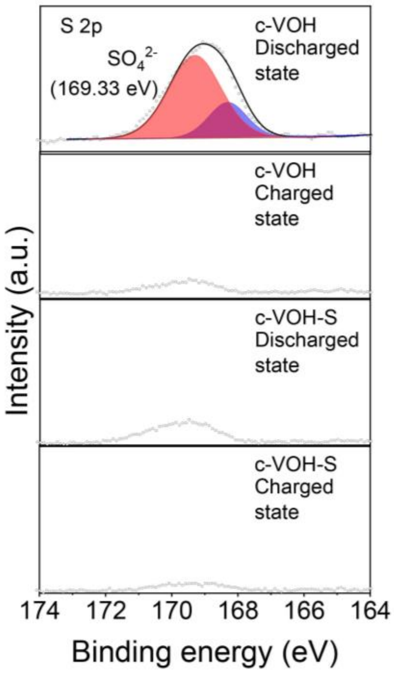

Figure S20. S 2p XPS spectra of the VOH cathodes after cycling in different electrolytes for two cycles at the fully discharged/charged state.

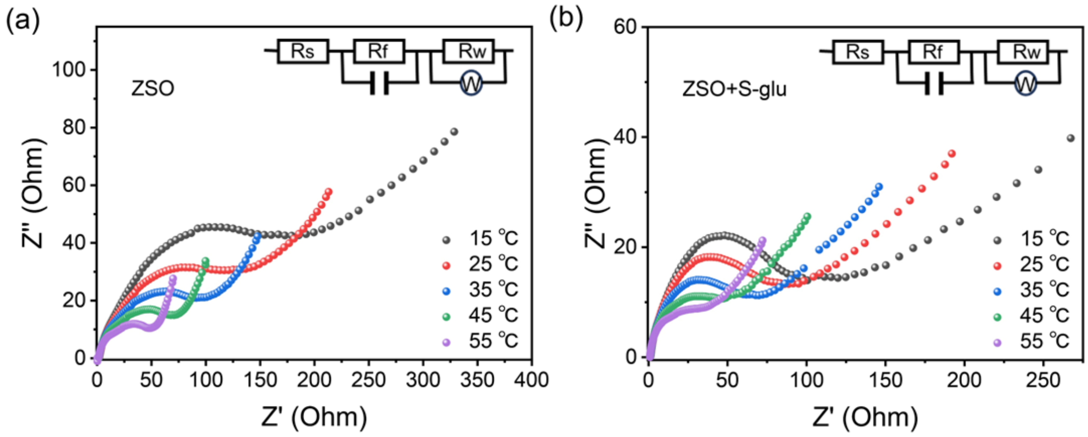

Figure S21. EIS spectra of the Zn||VOH cells after activation at different temperatures in ZSO electrolyte (a) without/(b) with S-glu additive. Inset is the fitting circuit diagram of the EIS spectra.

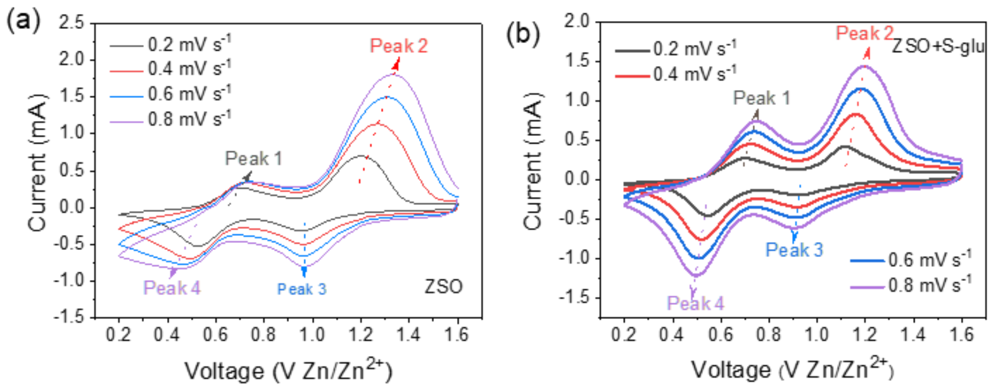

Figure S22. CV curves of the VOH cathode at different sweep rates in ZSO electrolyte (a) without and (b) with S-glu additive.

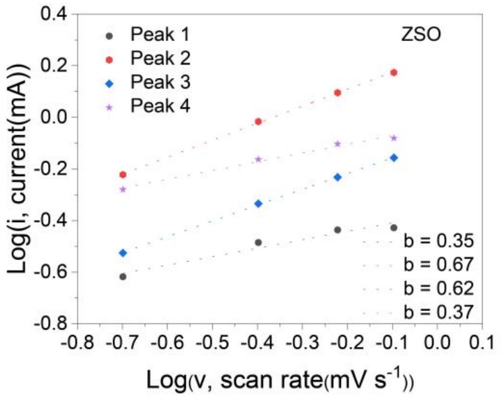

Figure S23. Calculated *b* values of the four peak currents according to the slope of log *i* vs log *v* in the CV curves for the ZSO system.

The relationship between the peak current (*i*) and scan rate (*v*) can be described by the following equation: $i = av^b$, where *a* and *b* are adjustable parameters. Correspondingly, $b = 0.5$ indicates a diffusion-controlled process, while $b = 1.0$ suggests a pseudocapacitive procedure. To determine the *b* values, the curves of log (*i*) *vs.* log (*v*) were plotted and linearly fitted to the redox peaks, in which the fitting slopes were equal to the *b* value.

As shown in Fig. 4g and Fig. S23, the *b* values of peak 1-4 were 0.72, 0.93, 0.74, and 0.85 in the S-glu-containing system, respectively, all exceeding the corresponding *b* values of the S-glu-free system. This means that the capacitive contribution takes the more proportion for the Zn^{2+} storage of VOH in the S-glu-containing electrolyte, thus leading to fast reaction kinetics.

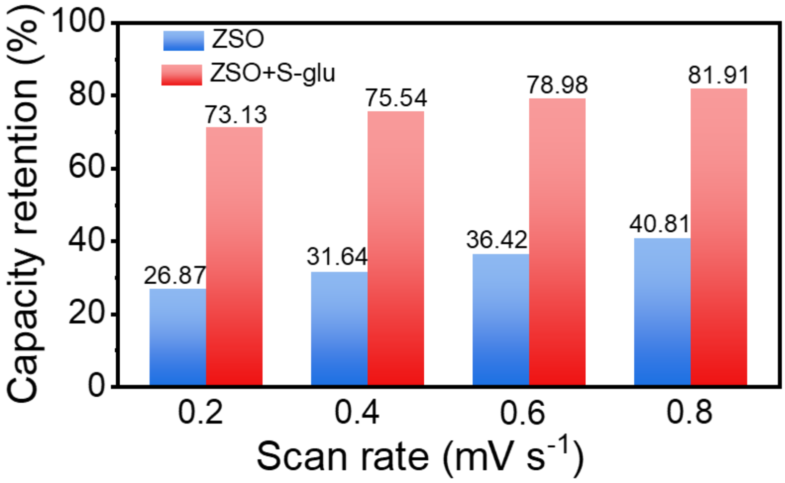

Figure S24. Contribution ratios of the capacitive capacities and diffusion-limited capacities of the VOH cathode in different electrolytes.

The pseudocapacitance and diffusion contribution can be separated and quantified based on the equation of $i = k_1v + k_2v^{1/2}$. When the k_1 and k_2 values are determined, the percentages of current controlled by capacitive effect (k_1v) and diffusion ($k_2v^{1/2}$) can be calculated for different scan rates.

When the scan rate increases from 0.2 to 0.8 mV s^{-1}, the capacitive contribution of the S-glu-containing cell increases from 71.13% to 81.91%, much higher than those of the S-glu-free counterpart (from 26.87% to 40.81%, Fig. S24), further implying that the introduction of the S-glu additive could promote the reaction kinetics.

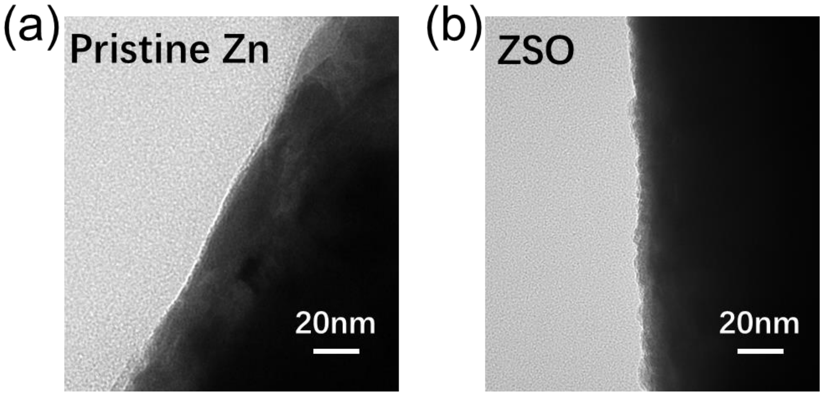

Figure S25. TEM image of the (a) pristine Zn anode and (b) Zn anode after 10 cycles in ZSO electrolyte under 2 mA cm^{-2}/2 mAh cm^{-2}.

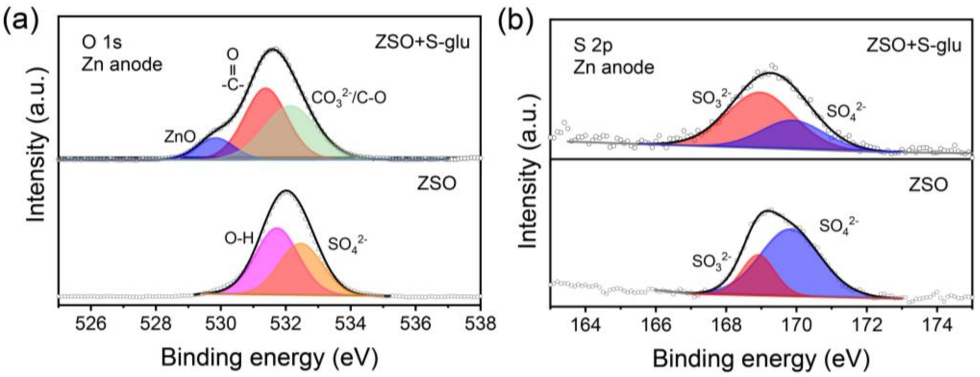

Figure S26. (a) O 1s and (b) S 2p XPS spectra of the Zn anodes after 10 cycles under 1 mA cm^{-2}/1 mA h cm^{-2} in different electrolytes.

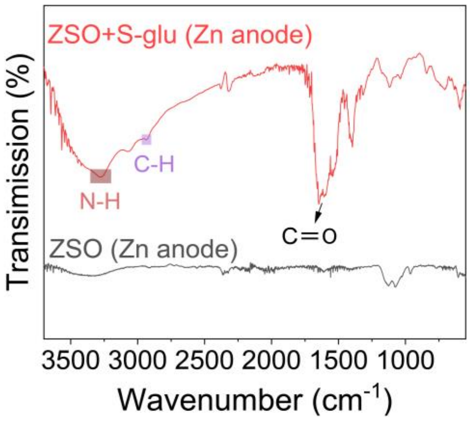

Figure S27. FTIR spectra of the Zn anodes after 10 cycles under 1 mA cm^{-2}/1 mA h cm^{-2} in different electrolytes.

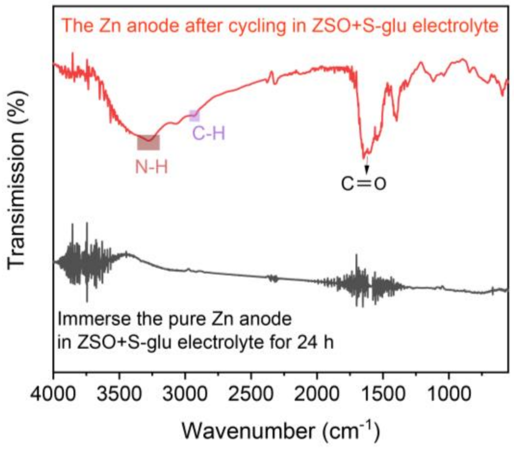

Figure S28. FTIR spectra of the Zn anodes after cycling and immersing in the ZSO+S-glu electrolyte. The FTIR spectrum of the Zn anode after soaking in the S-glu containing electrolyte do not show any organic vibration signal (e.g., C=O, C-H), suggesting that the anode EEI layers could not be formed via the simple contact between the electrode and the electrolyte.

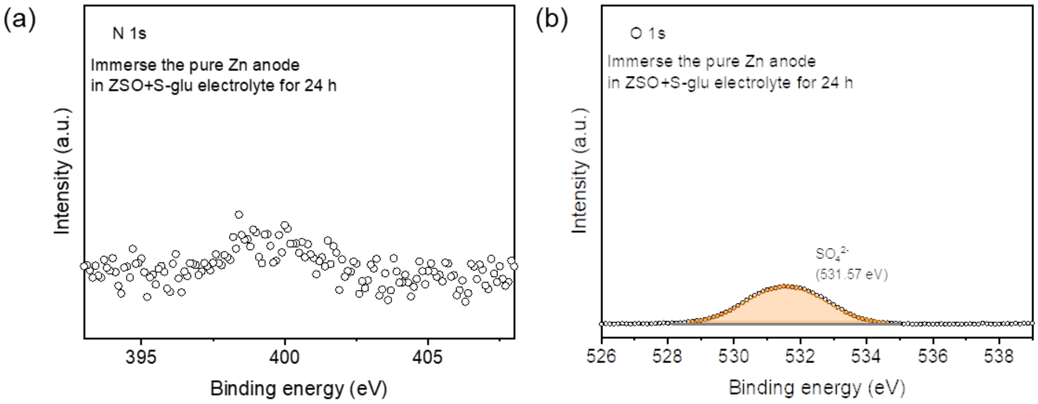

Figure S29. (a) N 1s and (b) O 1s XPS spectra of the Zn anode after immersing in ZSO+S-glu electrolyte for 24h. The O 1s XPS spectrum only showed the binding energies of SO_4^{2-} at 531.57 eV, and no signal was observed in the N 1s XPS spectrum, which suggest that no anode EEI layer was formed.

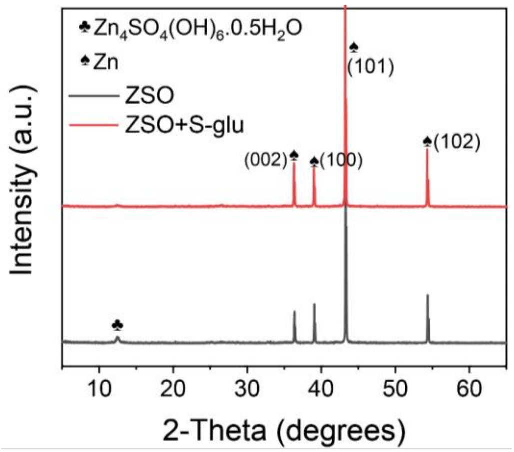

Figure S30. XRD patterns of the Zn anodes after 50 plating/stripping cycles in different electrolytes at 1 mA cm^{-2}/1 mA h cm^{-2}. The XRD pattern of the Zn anode cycled in the ZSO electrolyte with S-glu shows almost invisible sign of by-products $Zn_4SO_4(OH)_6{\cdot}0.5H_2O$.

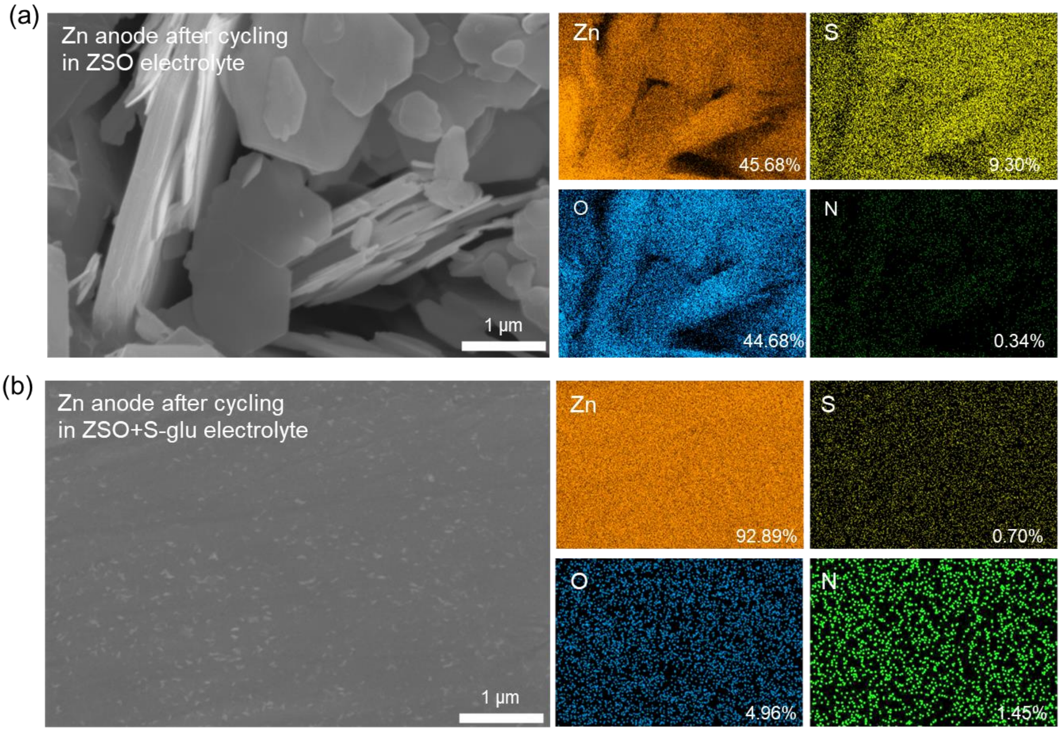

Figure S31. SEM image and corresponding energy dispersive spectrum with the elemental maps of Zn, O, S, and N of the Zn anode after cycling in ZSO electrolyte (a) without and (b) with S-glu additive for 10 cycles under 2 mA cm^{-2}, 2 mAh cm^{-2}. Here, it was found that a lot of S atoms were detected on the surface of Zn anode after cycling in ZSO electrolyte, suggesting the formation of by-product $Zn_4SO_4(OH)_6·0.5H_2O$, which is lined with the XRD results (Figure S26). Meanwhile, the presence of N on the surface of Zn anode after cycling in ZSO+S-glu electrolyte indicated the formation of PGA arising from the polycondensation of S-glu.

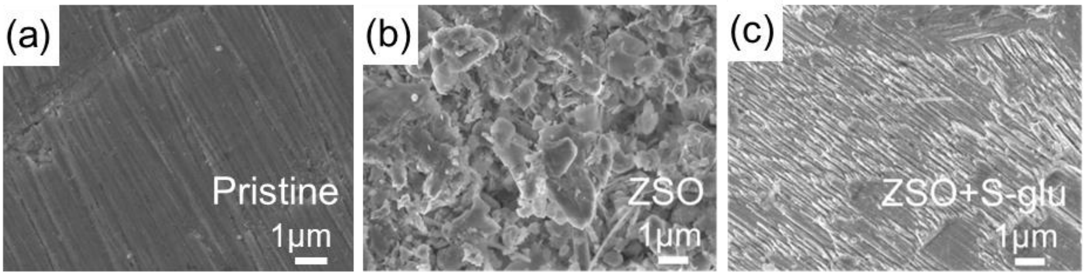

Figure S32. SEM images of the (a) pristine Zn anode, and Zn anodes after cycling in ZSO electrolyte (b) without/(c) with S-glu additive for 50 cycles at 1 mA cm^{-2}/1 mA h cm^{-2}.

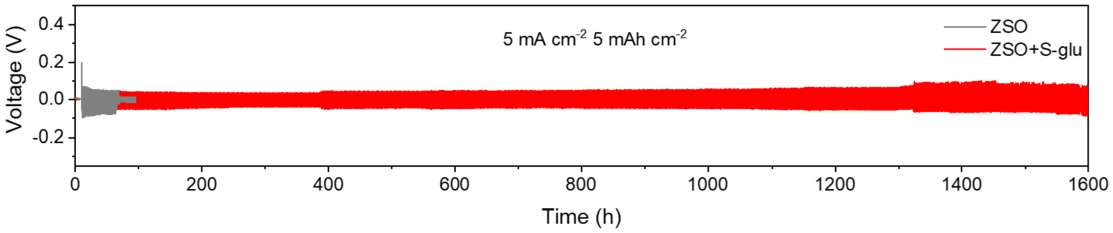

Figure S33. Cycling performance of the symmetric Zn‖Zn cells in ZSO electrolyte with/without S-glu additive at 5 mA cm^{-2}/5 mA h cm^{-2}.

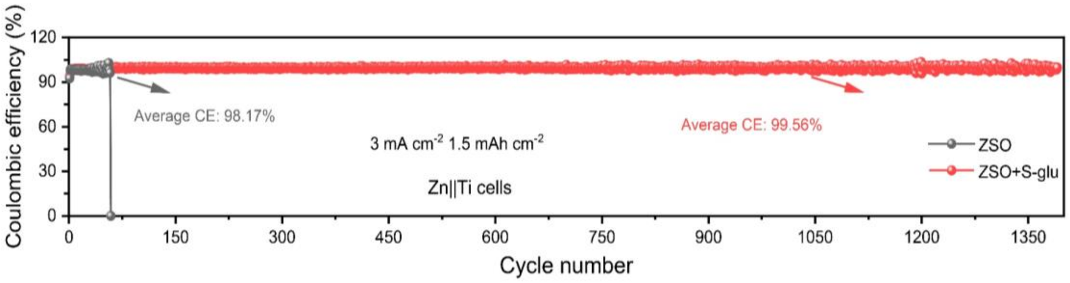

Figure S34. CE measurements of the Zn‖Ti cells at 3 mA cm^{-2}/1.5 mA h cm^{-2} in different electrolytes.

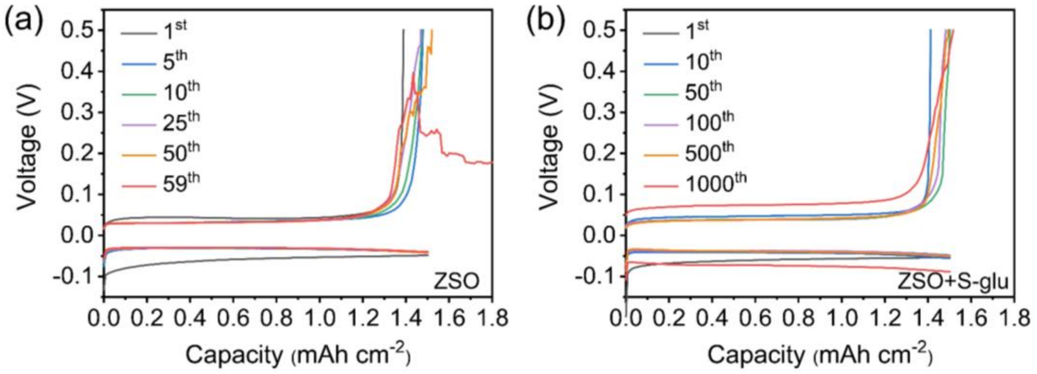

Figure S35. Voltage profiles of the Zn||Ti cells at various cycles in (a) ZSO and (b) ZSO+S-glu electrolyte at 3 mA cm^{-2}/1.5 mA h cm^{-2}.

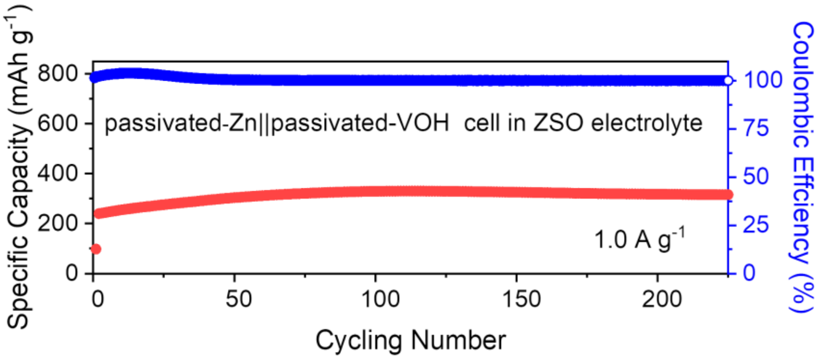

Figure S36. Cycling performance of the passivated-Zn||passivated-VOH cell in the ZSO electrolyte at 1 A g^{-1}. The passivated-Zn and passivated-VOH were collected from the Zn||VOH cell after 2 cycles in the ZSO+S-glu electrolyte at 0.2 A g^{-1}. The passivated electrodes were cleaned with alcohol and water before use to ensure a clean surface.

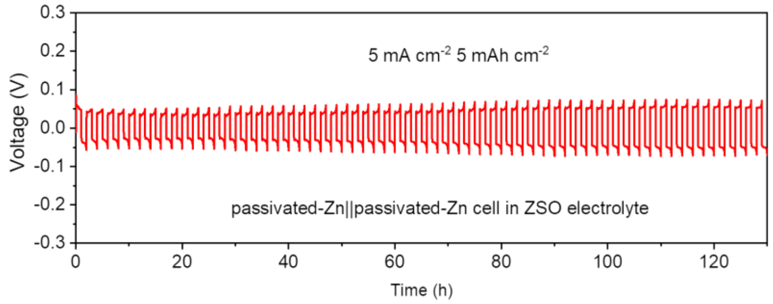

Figure S37. Cycling performance of the symmetric passivated-Zn||passivated-Zn cell in ZSO electrolyte at 5 mA cm^{-2}/5 mA cm^{-2}. The passivated-Zn was collected from the symmetric Zn||Zn cell after 10 cycles in the ZSO+S-glu electrolyte at 2 mA cm^{-2}/2 mAh cm^{-2} and then was cleaned with alcohol and water before use to ensure a clean surface.

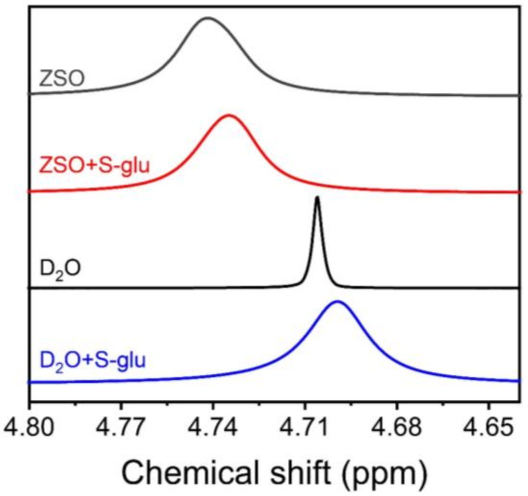

Figure S38. ^{1}H NMR spectra of H_2O, S-glu in H_2O, 2M ZSO electrolyte, and S-glu in 2M ZSO electrolyte.

The ^{1}H chemical shift of H_2O experiences an upshift with the addition of $ZnSO_4$ (4.706 ppm for H_2O and 4.743 ppm for ZSO electrolyte), indicative of decreased electronic density around H_2O due to the strong coordination between Zn^{2+} and H_2O. When S-glu was added into the ZSO electrolyte, the ^{1}H chemical shift moves to 4.735 ppm, implying some confined H_2O molecules in the solvated structure of Zn^{2+} were free again. This means that the introduction of S-glu additive could reduce the number of solvated water, which could reduce the activity of water and thus suppress water-related side reactions. Moreover, when only S-glu was contained in pure H_2O (H_2O+S-glu), the ^{1}H peak shifts to high field (4.699 ppm) compared with pure H_2O (4.706 ppm), indicating that the S-glu additive could also break the H-bond among water molecules, which is also helpful to suppress the activity of the electrolyte.[8] These results clearly manifest that the S-glu can change the solvation structure of Zn^{2+} and hydrogen bond network, which are beneficial to inhibiting water-related side reactions and thus improving the reversibility of Zn plating/stripping. The change the solvation structure of Zn^{2+} and hydrogen bond network also are the reason why the symmetric passivated Zn||passivated Zn cells with ZSO electrolyte cannot reach the cycle lifetime of symmetric pristine Zn||pristine Zn cells with ZSO+S-glu electrolyte.

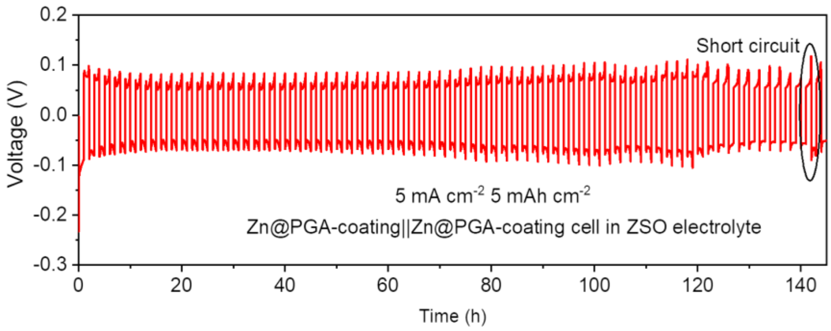

Figure S39. Cycling performance of the symmetric cell based on the PGA-coated Zn foils in the ZSO electrolyte at 5 mA cm^{-2}/5 mA cm^{-2}. To prepare the PGA-coated Zn anodes, a precise quantity of PGA was dissolved in a small amount of water, resulting in a viscous liquid with adhesive properties. This solution was then applied to the zinc sheet use a scraper, which was then dried in a vacuum oven at 80°C.

The symmetric cell comprising PGA-coated Zn anodes with the ZSO electrolyte gained a modest improvement in cycle performance compared to the Zn||Zn cell with the same electrolyte, but its performance still remains inferior to that of the Zn||Zn cells using the S-glu containing electrolyte. We aim to elaborate on the reasons for the inferior performance of symmetric Zn@PGA-coating||Zn@PGA-coating cell in the following aspects. (1) Such coating layers are prone to be detached and cannot easily tolerate the dynamic Zn plating/stripping process due to their poorer adhesion to Zn compared to the chemically bonded in situ EEI layer. (2) The in-situ polymer-inorganic EEI layer integrating high modulus of the inorganic component ($ZnSO_3$) with high toughness of the organic polymer ingredient (PGA) can realize long-term interfacial stability, which is not achievable with a simple poly(glutamic acid) coating.

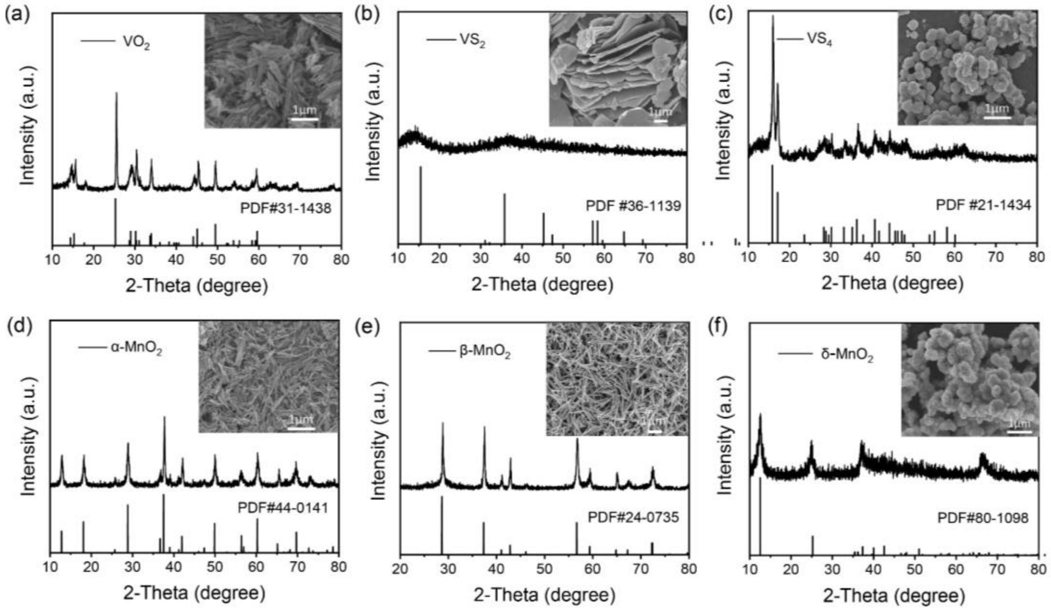

Figure S40. XRD patterns and SEM images (inset) of (a) VO_2, (b) VS_4, (c) VS_2, (d) α-MnO_2, (e) β-MnO_2, and (f) δ-MnO_2 material.

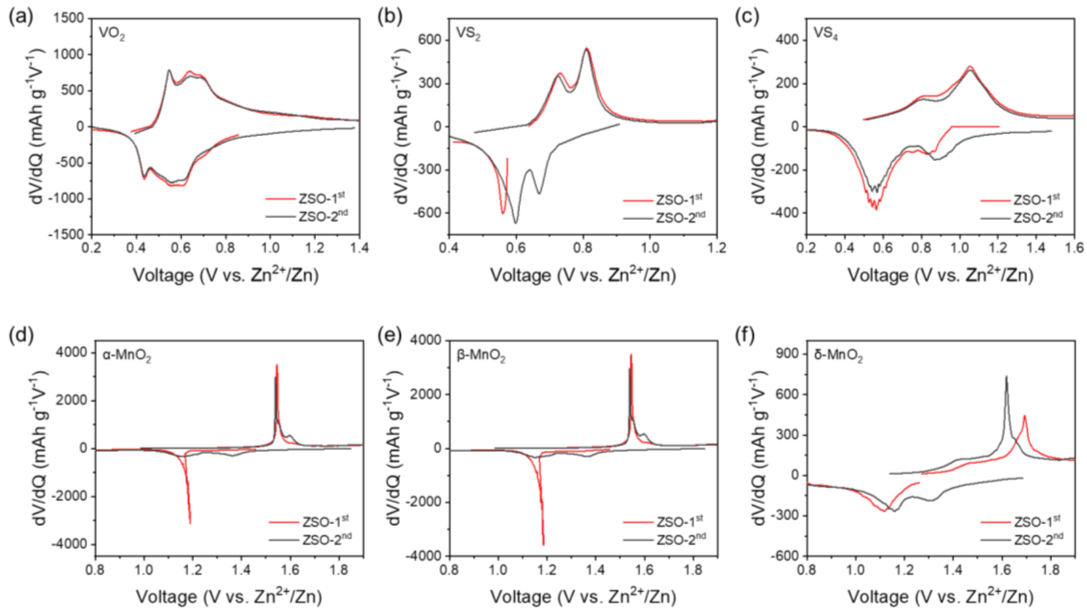

Figure S41. dQ/dV curves of initial two cycles of (a) VO_2, (b) VS_4, (c) VS_2, (d) α-MnO_2, (e) β-MnO_2, and (f) δ-MnO_2 in ZSO electrolytes under 0.5 A g^{-1}.

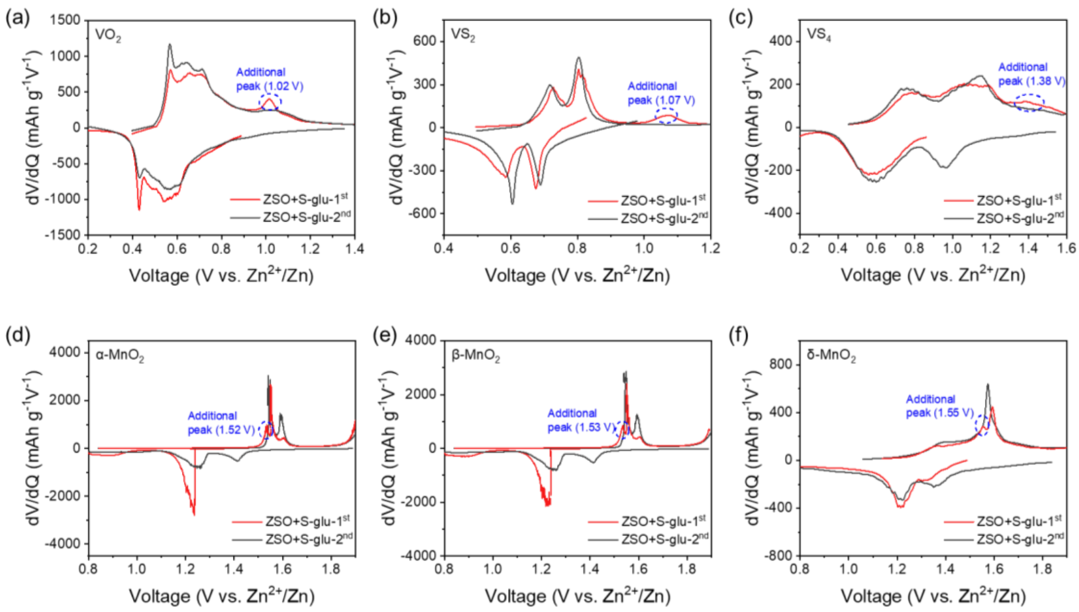

Figure S42. dQ/dV curves of initial two cycles of (a) VO_2, (b) VS_4, (c) VS_2, (d) α-MnO_2, (e) β-MnO_2, and (f) δ-MnO_2 in ZSO+S-glu electrolytes under 0.5 A g^{-1}. Compared with the cathode cycling in pure ZSO electrolyte (**Figure S32**), the additional peak related to the electro-polymerization of S-glu was observed in the initial dQ/dV plot of the S-glu containing cells, suggesting the formation of E-PGA film on the cathode materials.

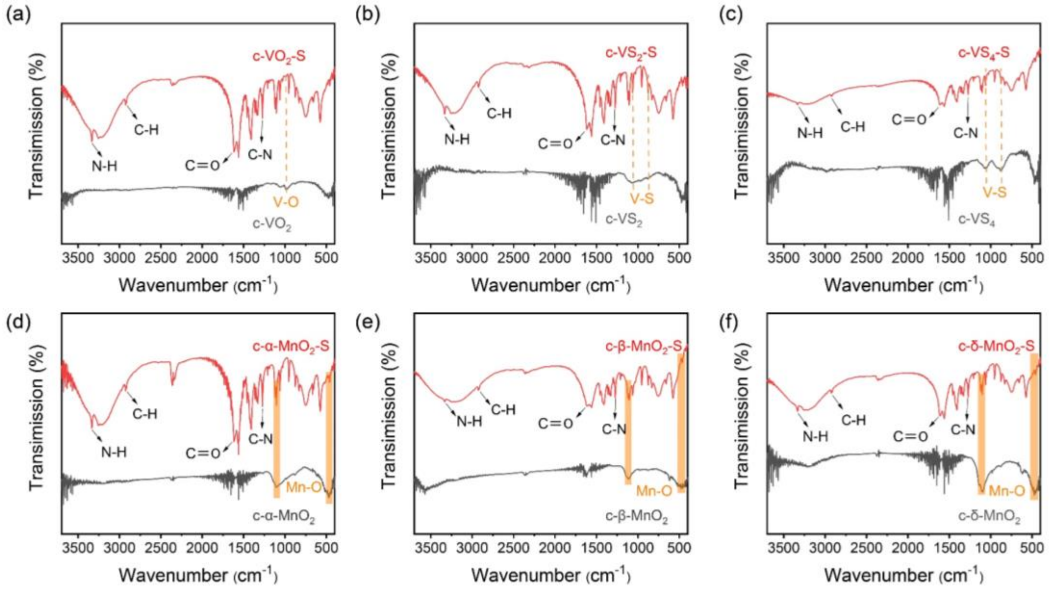

Figure S43. FTIR spectra of (a) VO_2, (b) VS_4, (c) VS_2, (d) α-MnO_2, (e) β-MnO_2, and (f) δ-MnO_2 after two cycles in different electrolytes under 0.1 A g^{-1}. The "c-cathode material" represents the cathode cycled in the ZSO electrolyte, while "c-cathode material-S" corresponds the one cycled in the S-glu containing electrolyte.

The FTIR spectra of these cathodes after cycling in ZSO with S-glu electrolyte exhibited the vibration stretching of N-H of secondary amino group at 3332-3334 cm^{-1}, the vibration stretching of N-H at 2924-2926 cm^{-1}, the typical peak of C=O stretching at 1615-1618cm^{-1}, and the signal of the vibration stretching of C-N at 1273-1274 cm^{-1}, which should be ascribed to the formation of PGA on the surface of cathodes[1]. In contrast, the cathodes after cycling in ZSO electrolyte only exhibited the typical V–O (994 cm^{-1}), V-S (1071 and 881 cm^{-1})[1-3], or Mn-O stretching (1108 and 474 cm^{-1})[4-6], suggesting that no EEI was formed on the cathode surface.

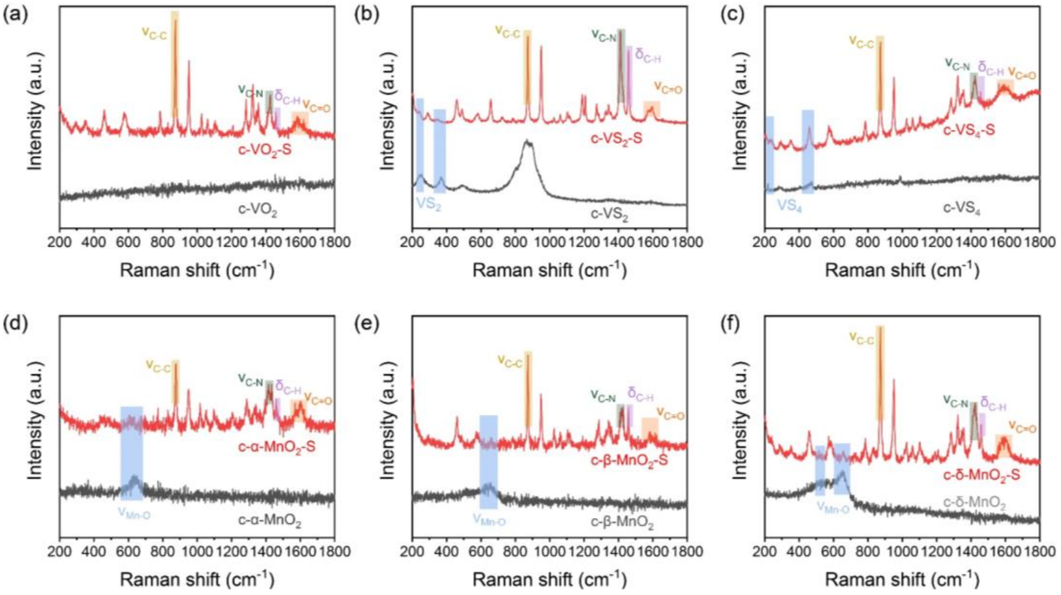

Figure S44. Raman spectra of (a) VO_2, (b) VS_4, (c) VS_2, (d) α-MnO_2, (e) β-MnO_2, and (f) δ-MnO_2 after two cycles in different electrolytes under 0.1 A g^{-1} in different electrolytes. The "c-cathode material" represents the cathode cycled in the ZSO electrolyte, while "c-cathode material-S" corresponds the one cycled in the S-glu containing electrolyte.

Different from the cathodes after cycling in ZSO electrolyte, the characteristic peaks at ~876, 1416, 1460, and 1599 cm^{-1} were detected for the cathodes after cycling in the S-glu containing electrolyte, which can be described as the C-C vibration, C-N vibration, C-H stretching, and C=O vibration, respectively, further proving the formation of PGA[7,8].

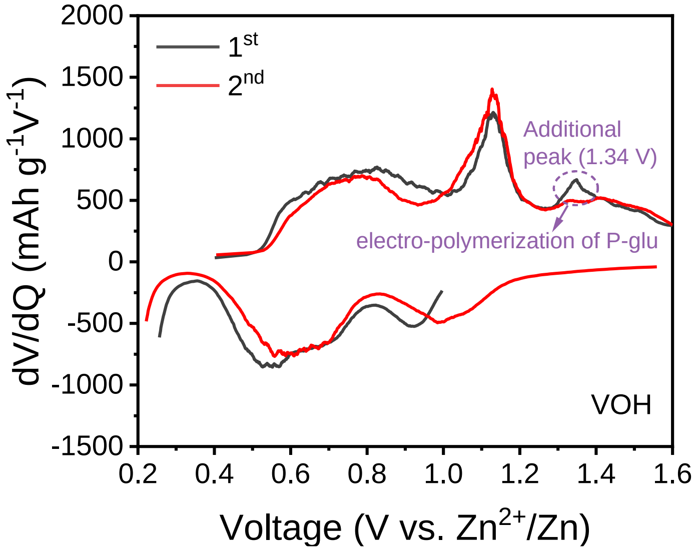

Figure S45. dQ/dV curves of initial two cycles of Zn||VOH cell at 0.2 A g^{-1} in the ZSO electrolyte with P-glu additive. An additional peak at 1.34 V related to the electro-polymerization of P-glu was observed in the initial dQ/dV plot, suggesting the formation of E-PGA film on the VOH cathode.

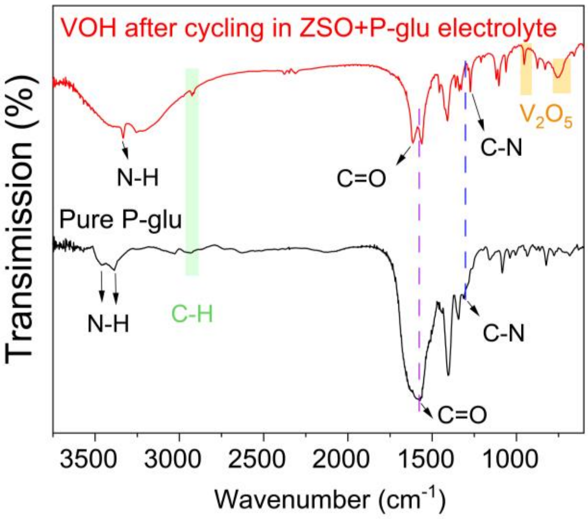

Figure S46. FTIR spectra of pure P-glu and VOH cathode after two cycles under 0.2 A g^{-1} in the ZSO electrolyte with P-glu additive. The FTIR spectrum of the VOH after cycling in ZSO+P-glu electrolyte exhibited the vibration stretching of N-H of secondary amino group (3334 cm^{-1}), the typical peak of C=O stretching (1617 cm^{-1}), and the signal of the vibration stretching of C-N (1276 cm^{-1}), which are slightly different from those observed in the FTIR spectrum of pure P-glu, ascribing to the formation of E-PGA.

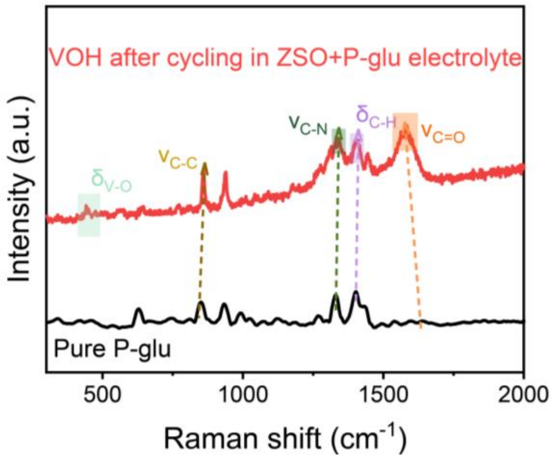

Figure S47. Raman spectrum of VOH cathode after two cycles in different electrolytes under 0.2 A g^{-1} in the ZSO electrolyte with P-glu additive.

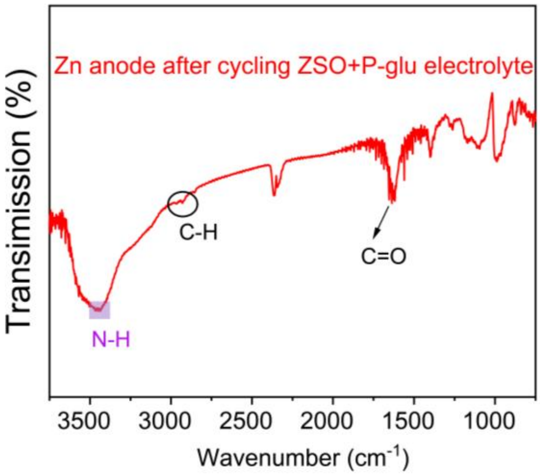

Figure S48. FTIR spectra of the Zn anodes after cycling in the ZSO+P-glu electrolyte. The FTIR spectrum of the Zn anode after cycling in the P-glu containing electrolyte shows the organic vibration signal of N-H, C-H, and C=O, suggesting that the anode EEI layer was formed *via* the electro-polymerization of P-glu.

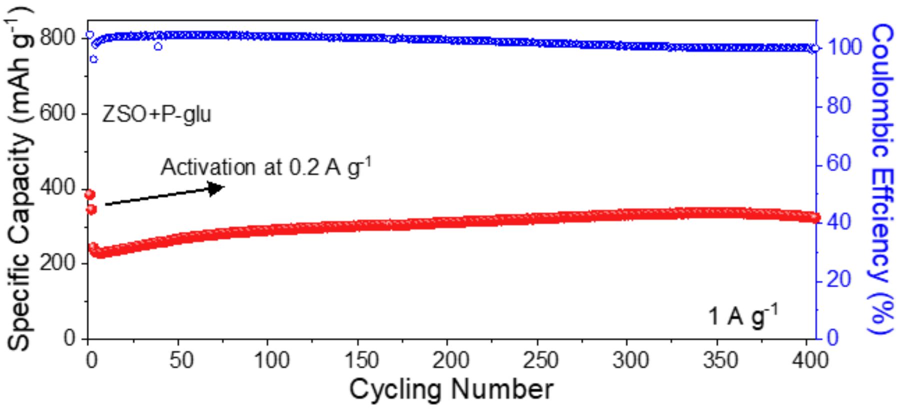

Figure S49. Cycling performance of Zn||VOH cell at 1.0 A g^{-1} in the ZSO electrolyte with P-glu additive.

The Zn||VOH cell with the P-glu containing electrolyte also displayed excellent cycle stability at 1.0 A g^{-1}, providing a high capacity of 328 mAh g^{-1} with nearly 100% Coulombic efficiency over 400 cycles. The improved performance can also be attributed to the protection of the in situ generated EEI layers.

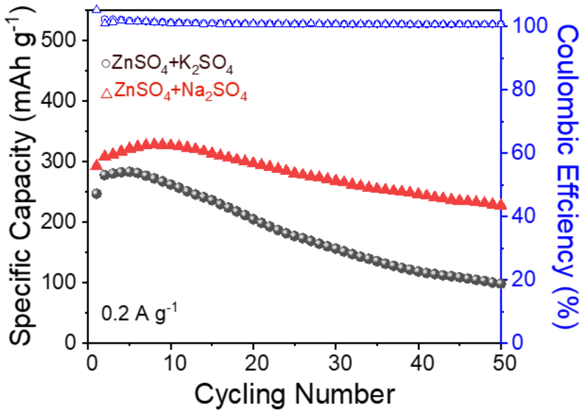

Figure S50. Cycling performance of Zn||VOH cell at 0.2 A g^{-1} in the ZSO electrolyte with 0.3 M KSO_4 and $NaSO_4$, respectively.

To investigate the effect of cation on the electrochemical properties, we introduced 0.3 M KSO_4 and $NaSO_4$ into a 2 M ZSO electrolyte and assembled Zn||VOH full cell. As shown in **Figure S50**, both full cells with the different electrolytes displayed poor cyclic stability, likely due to the depletion of active materials caused by the lack of an EEI layer. Therefore, compared with the absence of in situ EEI layer induced by glutamate anion, the influence of cations on battery performance is deemed negligible.

Table S1. Price of some commonly reported Zn-salts and solvents

Salt/solvent	$ZnSO_4$#	$ZnSO_4$	$ZnCl_2$#	$ZnCl_2$	$Zn(Ac)_2$#	$Zn(Ac)_2$
Price ($ kg^{-1})	0.35	37.22	1.05	44.71	2.53	19.12
Salt/solvent	$Zn(BF_4)_2$#	$Zn(BF_4)_2$	$Zn(OTf)_2$#	$Zn(OTf)_2$	$Zn(NO_3)_2$	$Zn(ClO_4)_2$
Price ($ kg^{-1})	7.03	25.03	42.18	421.52	58.77	1053.09
Salt/solvent	$Zn(TFSI)_2$	H_2O	EG	TEP	AN	TMP
Price ($ kg^{-1})	3676.69	0.1	0.5	14.7	21.99	28.2

Price information on these Zn-salts and solvents was mainly taken from https://www.aladdin-e.com (Aladdin, one of the major reagent suppliers in China), while the items marked with # (industrial grade) were obtained from https://b2b.baidu.com.

References

1 Li, Z.; Ganapathy, S.; Xu, Y.; Zhou, Z.; Sarilar, M.; Wagemaker, M., Mechanistic insight into the electrochemical performance of Zn/VO_2 batteries with an aqueous $ZnSO_4$ electrolyte. *Adv. Energy Mater.* **2019**, *9*, 1900237.

2 He, P.; Yan, M. Y.; Zhang, G. B.; Sun, R. M.; Chen, L. N., An, Q. Y.; Mai, L. Q., Layered VS_2 nanosheet-based aqueous Zn Ion battery cathode. *Adv. Energy Mater.* **2017**, *7*, 1601920.

3 Zhu, Q.; Xiao, Q.; Zhang, B.; Yan, Z.; Liu, X.; Chen, S.; Ren, Z.; Yu, Y., VS_4 with a chain crystal structure used as an intercalation cathode for aqueous Zn-ion batteries. *J. Mater. Chem. A* **2020**, *8*, 10761-10766.

4 Zheng, J.; Cao, Z.; Ming, F.; Liang, H.; Qi, Z.; Liu, W.; Xia, C.; Chen, C.; Cavallo, L.; Wang, Z. Alshareef, H. N., Preferred orientation of TiN Coatings enables stable zinc anodes. *ACS Energy Lett.* **2022**, *7*, 197-203.

5 Zhang, N.; Cheng, F.; Liu, J.; Wang, L.; Long, X.; Liu, X.; Li, F.; Chen, J., Rechargeable aqueous zinc-manganese dioxide batteries with high energy and power densities. *Nat. Commun.* **2017**, *8*, 405.

6 Han, M.; Huang, J.; Liang, S.; Shan, L.; Xie, X.; Yi, Z.; Wang, Y.; Guo, S.; Zhou, J., Oxygen defects in beta-MnO_2 enabling high-performance rechargeable aqueous zinc/manganese dioxide battery. *iScience* **2020**, *23*, 100797.

7 Tang, B.; Wang, J.; Hutchison, J. A.; Ma, L.; Zhang, N.; Guo, H.; Hu, Z.; Li, M.; Zhao, Y., Ultrasensitive, multiplex raman frequency shift immunoassay of liver cancer biomarkers in physiological media. *ACS Nano* **2016**, *10*, 871-879.

8 Zhang, Q.; Luan, J.; Fu, L.; Wu, S.; Tang, Y.; Ji, X.; Wang, H., The three-dimensional dendrite-free zinc anode on a copper mesh with a zinc-oriented polyacrylamide electrolyte additive. *Angew. Chem. Int. Ed.* **2019**, *58*, 15841-15847.
